# Using Digital Technology to Quantify Habitual Physical Activity in Community Dwellers With Cognitive Impairment: Systematic Review

**DOI:** 10.2196/44352

**Published:** 2023-05-18

**Authors:** Ríona Mc Ardle, Khalid Abdul Jabbar, Silvia Del Din, Alan J Thomas, Louise Robinson, Ngaire Kerse, Lynn Rochester, Michele Callisaya

**Affiliations:** 1 Newcastle University Newcastle United Kingdom; 2 University of Auckland Auckland New Zealand; 3 Monash University Victoria Australia

**Keywords:** dementia, cognitive dysfunction, physical activity, digital technology, wearable electronic devices, remote sensing technology, systematic review, community, wearables, cognitive impairment, support, clinicians, sensing

## Abstract

**Background:**

Participating in habitual physical activity (HPA) can support people with dementia and mild cognitive impairment (MCI) to maintain functional independence. Digital technology can continuously measure HPA objectively, capturing nuanced measures relating to its volume, intensity, pattern, and variability.

**Objective:**

To understand HPA participation in people with cognitive impairment, this systematic review aims to (1) identify digital methods and protocols; (2) identify metrics used to assess HPA; (3) describe differences in HPA between people with dementia, MCI, and controls; and (4) make recommendations for measuring and reporting HPA in people with cognitive impairment.

**Methods:**

Key search terms were input into 6 databases: Scopus, Web of Science, Psych Articles, PsychInfo, MEDLINE, and Embase. Articles were included if they included community dwellers with dementia or MCI, reported HPA metrics derived from digital technology, were published in English, and were peer reviewed. Articles were excluded if they considered populations without dementia or MCI diagnoses, were based in aged care settings, did not concern digitally derived HPA metrics, or were only concerned with physical activity interventions. Key outcomes extracted included the methods and metrics used to assess HPA and differences in HPA outcomes across the cognitive spectrum. Data were synthesized narratively. An adapted version of the National Institute of Health Quality Assessment Tool for Observational Cohort and Cross-sectional Studies was used to assess the quality of articles. Due to significant heterogeneity, a meta-analysis was not feasible.

**Results:**

A total of 3394 titles were identified, with 33 articles included following the systematic review. The quality assessment suggested that studies were moderate-to-good quality. Accelerometers worn on the wrist or lower back were the most prevalent methods, while metrics relating to volume (eg, daily steps) were most common for measuring HPA. People with dementia had lower volumes, intensities, and variability with different daytime patterns of HPA than controls. Findings in people with MCI varied, but they demonstrated different patterns of HPA compared to controls.

**Conclusions:**

This review highlights limitations in the current literature, including lack of standardization in methods, protocols, and metrics; limited information on validity and acceptability of methods; lack of longitudinal research; and limited associations between HPA metrics and clinically meaningful outcomes. Limitations of this review include the exclusion of functional physical activity metrics (eg, sitting/standing) and non-English articles. Recommendations from this review include suggestions for measuring and reporting HPA in people with cognitive impairment and for future research including validation of methods, development of a core set of clinically meaningful HPA outcomes, and further investigation of socioecological factors that may influence HPA participation.

**Trial Registration:**

PROSPERO CRD42020216744; https://www.crd.york.ac.uk/prospero/display_record.php?RecordID=216744

## Introduction

### Background

Approximately 50 million people are living with dementia worldwide, with numbers expected to increase to 82 million by 2030 [1]. There has yet to be a successful disease-modifying treatment for dementia. The World Health Organization (WHO) acknowledges the condition as a global public health priority [2] and recommends that people with dementia receive accessible, affordable person-centered support and care to maintain functional abilities and quality of life and remain living within the community [2]. People with mild cognitive impairment (MCI) should also be provided with such opportunities, particularly interventions focusing on modifiable lifestyle factors that could slow the progression of the disease [3,4]. Promoting habitual physical activity (HPA) may help to decelerate dependency and disability in people with cognitive impairment.

HPA refers to any movement produced by skeletal muscles that requires energy expenditure [5]. It does not always have to be planned, structured, or related to physical fitness (ie, exercise) but can be any physical activity such as walking or gardening. Supporting people with dementia and MCI to maintain their HPA may contribute to the WHO’s recommendations, with a recent meta-analysis suggesting that physical activity moderates the decline in cognitive abilities and reduces the risk of transitioning to more severe cognitively impaired states [6]. Participating in physical activity may also support functional abilities, reduce the progression of cerebrovascular pathology, decrease mortality risk, and attenuate behavioral and psychological symptoms of dementia such as depression and mood [7-9]. Conversely, physical inactivity is associated with an increased risk of comorbidities such as cardiovascular disease, which have knock-on effects in accelerating the course of disease and loss of functional independence. Given the potential health benefits, the first physical activity guidelines for older adults with MCI or subjective cognitive decline have recently been released, highlighting the growing clinical importance of this area for people with cognitive impairments [10].

However, prior research on people with cognitive impairment primarily focuses on exercise-based interventions, such as aerobic and anaerobic activities [11]. These may not be feasible for people with chronic health conditions, frailty, or those from low socioeconomic backgrounds who do not have access to appropriate interventional services [12]. People may not enjoy the types of exercise interventions offered, lack motivation, or feel uncomfortable or unconfident to participate [13]. Therefore, it may be more appropriate to consider all HPA that people with cognitive impairment participate in.

Capturing HPA behaviors in people with cognitive impairment can be challenging, as historically, this has relied on self-report methods, such as questionnaires. Self-report methods may provide inaccurate data, as they only provide a snapshot of an individual’s HPA and are susceptible to recall bias [14]. With technological advances such as wearable technology and ambient sensors [15-17], HPA can now be measured objectively and continuously, allowing a comprehensive approach that can capture the volume, intensity, pattern, and variability of HPA [18]. These HPA domains provide a framework to synthesize the current literature, consistent with a previous review quantifying HPA in aged residential care settings [19]. To truly understand the benefits of HPA, we must first summarize the current methods and metrics that have been used in the literature to capture HPA in people with cognitive impairment and how these might differ depending on the severity or type of cognitive impairment. Findings from this review will provide important insight into which methods and metrics are most common and feasible to characterize HPA in people with different degrees of cognitive impairment and will highlight how people with dementia and MCI may differ in discrete HPA metrics compared to normal aging. Given that ambulatory activities such as walking are the most accessible and common forms of HPA for older adults [20,21], this review will focus only on ambulatory physical activities.

### Aims and Objectives

The key aim of this review is to understand how HPA is currently measured and described in people with dementia and MCI in community-based settings and to quantify the volume, intensity, variability, and pattern of HPA in this population. To do this, this review has 4 core objectives, which are listed in Textbox 1.

**The 4 core objectives of this study.**

1. To report the digital methods and protocols used to assess habitual physical activity (HPA) in people with cognitive impairment, including the type of technology, device locations (eg, body or ambient), assessment periods, criteria for defining HPA, and participant compliance and acceptability regarding these methods
2. To identify the metrics used to describe HPA in people with cognitive impairment within a framework that includes the HPA domains of volume, intensity, pattern, and variability
3. To report differences in HPA between different levels of cognitive impairment and disease subtypes
4. To make recommendations for measuring and reporting HPA in people with cognitive impairment and guide future research directions in this area

## Methods

### Search Strategy

A total of 6 databases were used for this search: Scopus, Web of Science, Psych Articles, PsychInfo, MEDLINE, and Embase. Key terms for the search strategy are detailed in Multimedia Appendix 1, with information regarding the full electronic search strategy. An initial search was conducted in September 2020, with a follow-up search conducted on November 2, 2021. Therefore, research articles published before November 2021 were considered for this review. This review was preregistered on PROSPERO (CRD42020216744) and designed following the PRISMA (Preferred Reporting Items for Systematic Reviews and Meta-analyses) guidelines [22]. Multimedia Appendix 2 contains the completed PRISMA checklist.

### Selection Criteria

Table 1 shows the eligibility criteria for article selection in this review.

**Table 1:** Eligibility criteria for article selection in this review.

Factors	Inclusion criteria	Exclusion criteria
Language	Published in the English language	Published in a language other than English
Time frame	Published before November 2021	N/A^a^
Location/setting	Community-dwelling settings	Aged residential care settings, including supportive living, assisted living, residential aged care, nursing homes, and care homes
Topic	Studies reporting on quantifiable HPA^b^ metrics as derived from digital devices or other quantifiable technological methods	Studies not concerned with physical activity metrics Studies only concerned with physical activity programs, such as aerobic classes Intervention outcomes of activity interested in baseline data only Qualitative data Physical activity metrics derived from self-report measures
Population	People who have been diagnosed with dementia or MCI^c^ due to dementia-related causes.	Children Adults without diagnosed cognitive impairment Adults with undefined cognitive impairment People with cognitive impairment due to factors other than dementia-related causes (eg, depression)
Publication type	Peer-reviewed publications	Conference abstracts Posters Study protocols Reviews Meta-analyses Gray literature

^a^N/A: not applicable.

^b^HPA: habitual physical activity.

^c^MCI: mild cognitive impairment.

### Data Extraction

**Figure 1:**
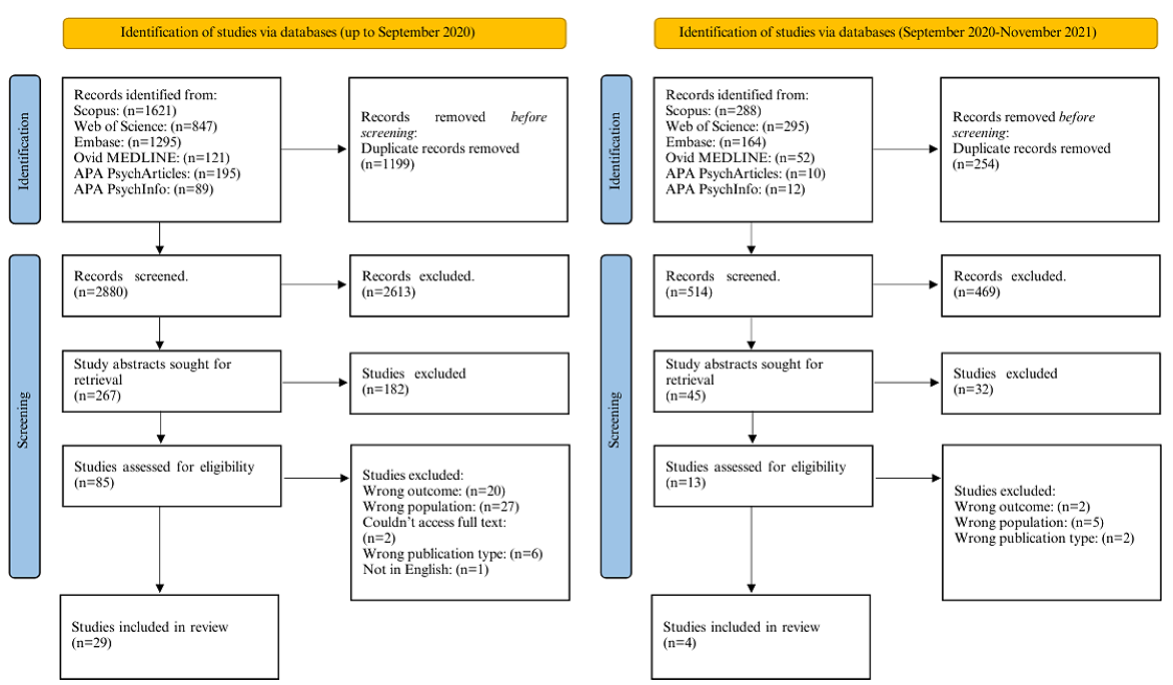
PRISMA (Preferred Reporting Items for Systematic Reviews and Meta-analyses) diagram demonstrating the search yield for this systematic review. APA: American Psychological Association.

All titles, abstracts, and full texts were independently screened by 2 reviewers (authors RMA and KJB) using Rayyan software developed by Ouzzani et al [23], with a third reviewer settling disagreements (author MC). Information about the decision-making process can be found in Figure 1. Data extraction forms were developed on Excel (Microsoft Corp) by author RMA and refined in consultation with 2 other authors (MC and KJB). Data were extracted from all eligible articles, with key measures of interest as follows: (1) diagnosis and diagnostic criteria applied; (2) method of HPA assessment (eg, technology, device location, time period, and criteria used to characterize HPA); (3) HPA metrics assessed and their values (eg, volume, intensity, pattern and variability metrics; see Table 2 for definitions); and (4) main finding of the articles in respect to HPA. A quality assessment was conducted independently by 2 reviewers (authors RMA and KJB) using the National Institute of Health Quality Assessment Tool for Observational Cohort and Cross-sectional Studies [24]. The quality assessment was adapted for this review by removing questions relating to the measurements of exposures of interest and by adding the following question: “Were clinical diagnostic criteria and severity ratings for dementia reported and adhered to?” This adapted version has previously been used in similar reviews [19,25]. Average scores determined the overall quality of each study.

**Table 2:** Definitions and prevalence of HPA^a^ metrics captured in this systematic review.

Physical activity characteristic		Papers assessing HPA metrics, n (%)	Description
**Volume of physical activity**		73 (24)	Time spent in physical activity for a specified time frame (eg, day, week). May also be described as the duration of physical activity
	Steps per day	42 (14)	Number of steps taken per day, usually averaged over a specified period
	Minutes/hours/percentage of time per day/week spent active/walking	27 (9)	Time spent in overall physical activity, dependent on criteria applied and methodology used to capture data
	Number of walking/active bouts/episodes	18 (6)	A walking/activity bout is defined as any period of continuous walking or ambulation. Different studies employ different thresholds for what they consider the start and end of a bout.
	Activity counts per day/hour/minute	15 (5)	A common unit of measurement for accelerometers. This is a derived score or unit that is dependent on the accelerometer used, as different devices process raw data in different ways
	Vector magnitude of counts per minute	6 (2)	Vector magnitude is a single triaxial composite metric that measures physical movement in the mediolateral, anteroposterior, and vertical axes.
	Distance moved per day	3 (1)	Units of distance (kilometers or miles) moved per day, averaged over a specified time frame
	Exercise per day	3 (1)	Defined as MET^b^ × hours + calories consumed = exercise × bodyweight × 1.05
	Outdoor time per day	3 (1)	Time spent outdoors moving per day taking steps, averaged over a specified period
**Intensity of physical activity**		46 (15)	The rate or magnitude in which physical activities are performed, indicating the metabolic demand of the activity
	Moderate-vigorous physical activity	15 (5)	Physical activity that is strenuous enough to burn >3 times as much energy (ie, >3 METs) per minute as you do when resting. The characterization of this metric depends on the criteria and methodology applied to the study.
	Light/low-intensity physical activity	15 (5)	Physical activity that burns 1.6-3 times as much energy (ie, <3 METs) per minute as you do when resting (eg, self-care activities, light domestic duties). The characterization of this metric depends on the criteria and methodology applied to the study.
	Moderate-intensity physical activity	12 (4)	Physical activity that is strenuous enough to burn 3-6 times as much energy (ie, 3-6 METs) per minute as you do when resting (eg, brisk walking, heavy household activities). The characterization of this metric depends on the criteria and methodology applied to the study.
	Physical energy expenditure per day	6 (2)	Energy expenditure is the amount of energy required to carry out physical functions, such as breathing, or exercising, measured in kilocalories. Physical energy expenditure is the number of calories you burn when active.
	Vigorous activity	6 (2)	Physical activity that is strenuous enough to burn >6 times as much energy (ie, >6 METs) per minute as you do when resting (eg, jogging, hiking, cycling). The characterization of this metric depends on the criteria and methodology applied to the study.
	Time spent in/number of 60+ second walking bouts	6 (2)	Time spent moving continuously for over 60 seconds at a time
	Total energy expenditure per day	3 (1)	Energy expenditure is the amount of energy required to carry out physical functions, such as breathing, or exercising, measured in kilocalories. Total energy expenditure is the number of calories you burn per day.
	Calories per minute during walking/activity	3 (1)	Energy expenditure per minute burned during walking activities
	Physical activity level	3 (1)	A way to express a person's participation in daily physical activity as a number, with definitions depending on the study (eg, relative EE^c^ to basal metabolic rate for all behaviors)
	Very light physical activity	3 (1)	Considered to be physical activity within the cutoff of 145-274 counts per minute within a single study
	MET minutes active per day/week	3 (1)	A MET minute is the amount of energy you expend during a minute while resting. Depending on the intensity at which you are performing physical activity, you can burn more than 1 MET minute per minute.
	Total movement intensity (g)	3 (1)	Mean vector magnitude of dynamic acceleration per day for total behavior, expressed relative to gravitational acceleration by the unit g (m/s^2^)
	Physical Activity Ratio	3 (1)	Relative energy expenditure to basal metabolic rate of activity
	Mean 10+ minute bouts per day	3 (1)	Time spent continuously moving for over 10 minutes
	Light-moderate physical activity	3 (1)	Considered to be physical activity within the cutoff of 274-597 counts per minute within a single study
	Brisk walking time	3 (1)	Time spent walking at a brisk pace
	1-minute peak cadence	3 (1)	Steps per minute recorded for the highest single minute in a day
**Pattern of physical activity**		42 (14)	Refers to the number of sessions and distributions of physical activity per day/week
	Mean bout length	15 (5)	A walking/activity bout is defined as any period of continuous walking or ambulation. Different studies employ different thresholds for what they consider the start and end of a bout. Mean bout lengths refer to how long walking/activity bouts are on average across a specified period.
	Alpha	9 (3)	Refers to the distribution of ambulatory/active/walking bouts according to their duration, related to the power law distribution. A large alpha score indicates that people are taking proportionally more short bouts than long (ie, the distribution of bouts is derived from a greater proportion of shorter bouts).
	Daytime activity	9 (3)	Characterization of physical activity during daytime hours, as determined by each study
	Night-time activity	9 (3)	Characterization of physical activity during nighttime hours, as determined by each study
	Afternoon activity	3 (1)	Characterization of physical activity during afternoon hours, as determined by each study
	Evening activity	3 (1)	Characterization of physical activity during evening hours, as determined by each study
	Mean 24-hour wavelet variance	3 (1)	A method of decomposing a time-varying signal at multiple resolutions employed to examine differences in 24-hour activity variance over time within a group. This reflects daily variance.
	Longest bout length duration	3 (1)	The longest duration of a period of continuous walking/ambulation
	Peak activity	3 (1)	The maximum activity per minute during waking hours
	Time of peak activity	3 (1)	The time at which the peak activity minute occurred
	Relative amplitude	3 (1)	The normalized difference between the most active 10-hour period in a 24-hour cycle in relation to the uninterrupted least active 5-hour period. Higher values indicate a higher amplitude, which means there are greater differences between daytime activity and nighttime rest, therefore a stronger rhythm.
**Variability of physical activity**		27 (9)	Refers to changes in physical activity metrics (eg, consistency/inconsistency, regularity/irregularity) either within-person or group and over time.
	Variability of bout length (S^2^/COV^d^)	12 (4)	Refers to the “within-person” variability of ambulatory/activity/walking bout lengths. Calculated either via maximum likelihood technique (S^2^) or by calculating the ratio of the standard deviation to the mean and showing variability in relation to the mean of the population (COV).
	Day-to-day variability	3 (1)	Examination of interday reliability between different days
	Hour-to-hour variability	3 (1)	Examination of interhour reliability between different hours
	COV of daily activity	3 (1)	Examining variability in physical activity by calculating the ratio of the standard deviation to the mean and showing variability in relation to the mean of the population (COV)
	Root mean square difference	3 (1)	A measure of physical activity complexity. The standard deviation of all minute-to-minute physical activity intervals during waking hours. Can indicate the extent to which a person’s activity over a period deviates from a flat, nonvarying rhythm
	Intraindividual variability	3 (1)	A measure of consistency/inconsistency in physical activity
	Intradaily variability	3 (1)	The degree of fragmentation of periods of rest and activity, with higher values indicating a more fragmented rhythm
	Intradaily stability	3 (1)	The regularity in the rest-activity rhythm over days, with lower scores indicating a lack of rhythm and higher scores indicating a stable rhythm

^a^HPA: habitual physical activity.

^b^MET: metabolic equivalent.

^c^EE: energy expenditure.

^d^COV: coefficient of variance.

### Data Synthesis

Narrative data synthesis was conducted to answer our research aims. This primarily considered the key outcomes of this review—the methods and metrics used to assess and characterize HPA in people with cognitive impairment and significant differences in HPA metrics across the cognitive spectrum. To support data interpretation and provide consistency across the literature, we adopted the same HPA framework as Mc Ardle et al [19] in their review of HPA assessment in aged care facilities, which groups HPA metrics into 4 domains: volume, intensity, pattern, and variability of HPA. Due to significant heterogeneity across the methods, protocols, metrics, and populations in the studies included in this review, a meta-analysis was deemed inappropriate.

## Results

### Search Yield

The initial search conducted in September 2021 identified 2880 papers (Figure 1). The updated search strategy conducted between September 2020 and November 2021 identified 514 articles (Figure 1). A total of 33 articles were included in this review following data extraction. All papers were published between 2008 and 2021.

### Study Characteristics

Multimedia Appendix 3 contains information relating to all the study characteristics and key results. Studies took place in Germany (n=7, 21%), the United States (n=6, 18%), the United Kingdom (n=5, 15%), Japan (n=4, 12%), the Netherlands (n=4, 12%), Belgium (n=2, 6%), Israel (n=2, 6%), Italy (n=2, 6%), Hong Kong (n=2, 6%), Taiwan (n=1, 3%), France (n=1, 3%), Canada (n=1, 3%), Brazil (n=1, 3%), Singapore (n=1, 3%), Australia (n=1, 3%), and Norway (n=1, 3%). One (3%) study did not specify which country it took place in. The sample size of participants with cognitive impairment ranged between 7 and 323 across all studies, with mean age ranging from 63 to 89 years. Regarding participants with cognitive impairment, 61% (n=20) of studies reported ≥50% of participants as female. Only 3 (9%) studies reported the ethnicity of participants with cognitive impairment; in all 3 (100%) studies, >85% of the participants were White. Most studies were cross-sectional (n=20, 61%), with 11 (33%) studies using baseline data from randomized controlled trials/interventions and 2 (6%) being feasibility/pilot studies. There were no longitudinal observational studies.

Levels of cognitive impairment described included MCI (n=13, 39%), mild dementia (n=2, 6%), mild-moderate dementia (n=5, 15%), unspecified level of dementia (n=12, 36%), a mix of MCI and dementia (n=2, 6%), and unspecified level of cognitive impairment (n=2, 6%). Of the 12 (36%) studies that specified dementia disease subtypes, all (n=12, 100%) reported participants with Alzheimer disease; 25% (n=3) reported participants with dementia with Lewy bodies, vascular dementia, or MCI/dementia due to Parkinson disease; 17% (n=2) reported participants with frontotemporal dementia, mixed dementia, or amnestic MCI; and 8% (n=1) reported participants with early-onset dementia, nonamnestic MCI, or Korsakoff syndrome. Moreover, 28 studies (85%) explicitly described procedures to characterize cognitive impairment (eg, clinician review, cognitive score thresholds), and 64% (n=18 studies) of these used validated diagnostic criteria (eg, [26-29]). Multimedia Appendix 4 reports on the quality of the studies.

### Measuring Physical Activity in Community Dwellers With Cognitive Impairment

**Figure 2:**
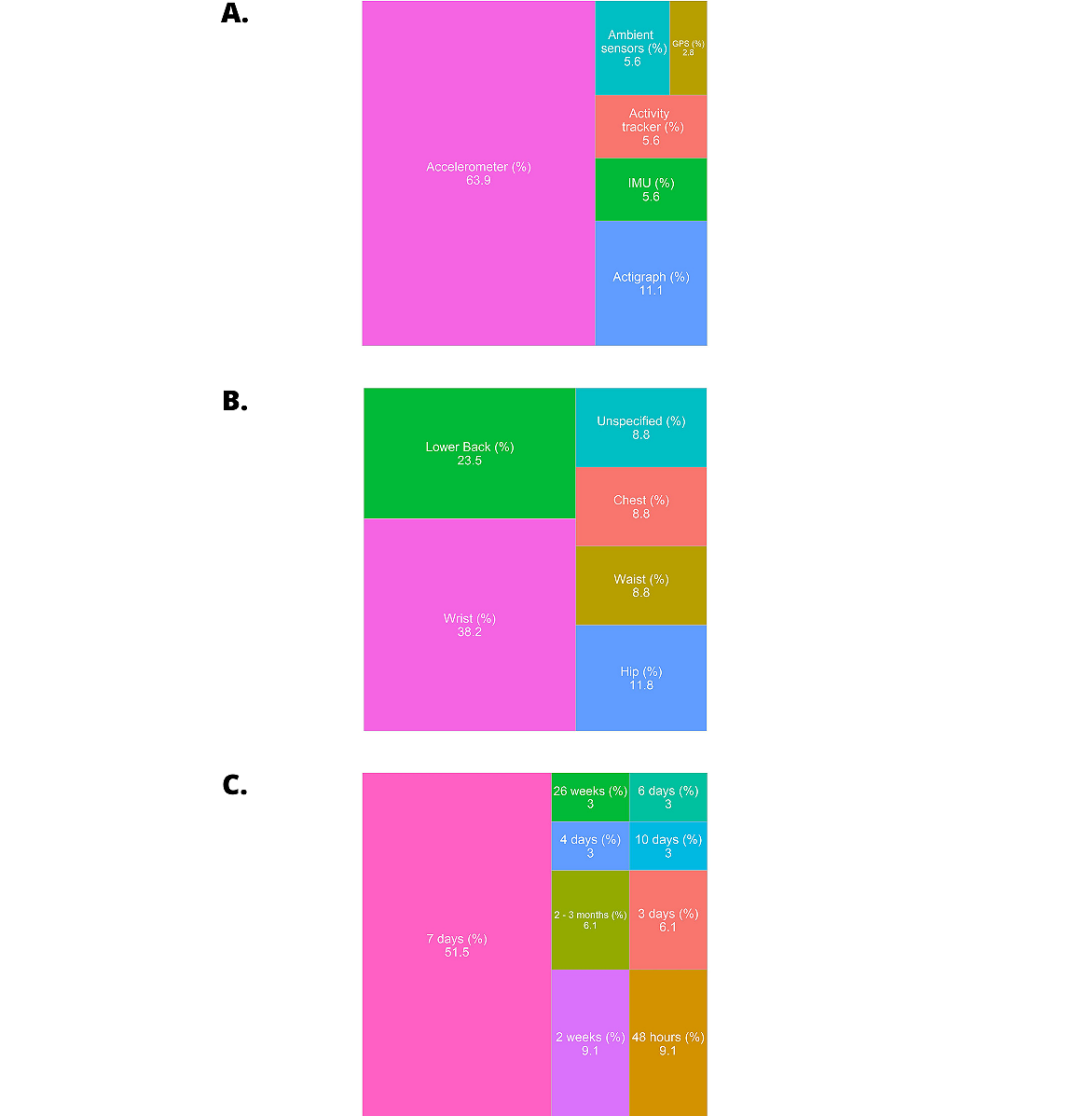
Methods and protocols used across studies included in this review, including devices, device locations, and data collection time periods. (A) Digital devices used to capture physical activity metrics; (B) placement of wearable devices on the body; (C) habitual physical activity collection time period. IMU: inertial measurement unit.

Out of 33 studies, 31 (94%) employed wearable technology to measure HPA in people with cognitive impairment, and 2 (6%) used ambient home-based sensors. The most popular device used was an accelerometer (n=23, 70%), while the most common device location was the wrist (n=13, 39%) followed by the lower back (n=8, 24%). Most study protocols asked participants to wear/use the device for seven days (n=17, 52%). Figure 2 provides further details regarding the measurement of HPA.

A total of 14 (42%) papers addressed reasons for loss of data and issues with adherence to protocol, including technical issues (n=5, 36%) [30-34], participants removing or refusing to wear devices (n=6, 43%) [30,33,35-38], insufficient data collected (n=8, 57%; <10 hours per day for 8/14 days [39]; <80% daily wear time [40]; <3 days [31]; <10 hours per day for at least 3 days [41]; <7 days [15]; <6 consecutive days [33]; incomplete recorded days [34,40]), lost devices (n=2, 14%) [15,33], organizational issues (n=2, 14%) [15,33], forgetting to wear the device (n=1, 7%) [42], and hospitalization during the data collection period (n=1, 7%) [38].

Only 1 (3%) study reported acceptability information, with 83% of participants in the study by Rawtaer et al [43] finding the use of multiple remote monitoring devices, including a wrist-worn sensor, acceptable, saying it was comfortable and enjoyable to wear but inconvenient to charge or wear out of the home in some cases. No studies considered levels of cognition or dementia subtype when reporting compliance or acceptability.

Table 2 provides definitions of all HPA metrics included in this review. Of the 33 studies included, 24 (73%) reported HPA metrics relating to volume, 15 (46%) relating to intensity, 14 (42%) relating to pattern, and 9 (27%) relating to the variability of HPA.

### Volume

#### MCI Measurement

Of the 13 (39%) studies reporting HPA in MCI, 8 (62%) characterized volumes [32,34,40,41,43-47]. The most common volume metric was steps per day (n=5, 39%), with mean/median averages ranging between 3407 and 12,256 steps [40,41,43,44,46,47]. Two (15%) studies reported time spent walking per day, with a mean range between 113 and 150 minutes [41,44]. The percentage of the day spent active ranged between 3.9% and 8.7% across 2 (15%) studies [40,44], with the same studies reporting a range between 261 and 490 walking bouts per day. Another 2 (15%) studies reported the mean percentage of time spent active, which ranged between 8.69% and 21% [40,45].

#### Global Cognitive Impairment/Mixed Groups

Of the 2 studies that measured HPA in participants with global cognitive impairment [39,48], both (n=2, 100%) reported mean steps per day, ranging from 6654 to 6721.

#### Dementia

A total of 8 studies measured the volume of HPA in groups of people with dementia of unspecified severity [33,37,40,42,49-52]. Among them, 2 (25%) studies reported steps per day, with means ranging between 1509 and 2362 steps [40,49]. Moreover, 2 (25%) reported percentage of time spent walking per day, ranging between 1.8% and 4.9% [37,40], and 2 (25%) studies captured activity counts per day, ranging between 2.11 × 10^5^ and 2.21 × 10^5^.

### Intensity

#### Overview

A total of 9 (27%) studies reported their quantification of different levels of HPA intensity, among which 1 (11%) reported very light physical activity, with counts per minute (CPM) ranging between 145 and 274 [50]. Moreover, 6 (66%) studies quantified light physical activity differently, including a metabolic equivalent (MET) ranging between 1.6 and 3 [40], 1.5 and 2.9 [53], and 1 and 3 [54] and CPM between 150 and 2689 [52], 100 and 1951 [55], and 100 and 2019 [34]. Another 4 (44%) studies characterized moderate physical activity, including an MET between 3 and 6 [40,53] and CPM between 1952 and 5742 [55] and 2020 and 5998 [34]. The studies report light-moderate physical activity as having a CPM between 274 and 597 [50]. Additionally, 4 (44%) studies quantified moderate-vigorous physical activity (MVPA), including >3 MET [45], >597 CPM [50], >6367 CPM [41], and >2690 CPM [52]. Another 3 (33%) studies characterized vigorous physical activity differently, including ≥6 MET [40], CPM between 5743 and 9498 [55], and CPM ≥5999 [34]. Only 1 (11%) study reported very vigorous physical activity as ≥9798 CPM [55].

#### MCI Measurement

Of the 15 (46%) studies included that reported intensity metrics, 9 (60%) were in MCI groups. Physical activity–related energy expenditure was most commonly assessed (n=3, 33%), with means averaging between 114 and 775 kcal/day [34,40,46], while 1 (11%) study also reported total energy expenditure as 2572 kcal/day on average [40]. Moreover, 2 (22%) studies reported the percentage of the day spent in light physical activity, with means ranging between 6% and 25%, along with percentages spent in moderate intensity HPA (3%-6%) and vigorous intensity HPA (0%-0.07%) [34,40]. Only 1 study reported participants spending 3% of their time in MVPA [34], while 2 (22%) reported a mean/median range between 9 and 24 minutes spent in MVPA per day [41,53]. Another study (n=1, 11%) reported 324 to 353 minutes spent in light HPA per day [53].

#### Global Cognitive Impairment/Mixed Groups

Only 1 (3%) study reported an average of 31.7 (SD 21.2) minutes per day spent in MVPA in a group with global cognitive impairment [48]. No other studies reported the intensity of HPA in this subgroup.

#### Dementia

We identified 5 (15%) studies that described the intensity of HPA in dementia of unspecified disease severity [40,42,50,55,56], with 2 (40%) reporting a mean range of 5% to 51% of time spent in light physical activity and 2% to 10% of time spent in moderate physical activity [40,55].

### Pattern

#### MCI Measurement

We found 5 (15%) studies that reported metrics relating to the pattern of physical activity in MCI [32,34,40,44,57]. Among them, 2 (40%) studies reported mean walking bout length, which ranged from 11 to 28 seconds [40,58].

#### Global Cognitive Impairment/Mixed Groups

We found 1 (3%) study that reported on the pattern of physical activity in groups of people with mixed cognitive impairment encompassing MCI and dementia [38]. That study described a 16.3-second (SD 3 seconds) average walking bout length and a mean alpha score of 1.640.

#### Dementia

In terms of dementia, 7 (21%) studies reported HPA pattern metrics in groups of people with dementia of unspecified severity [31,33,37,40,51,52,57]; 3 (43%) of these specified Alzheimer disease [31,51,57] and 1 (14%) specified Parkinson disease dementia [40]. The mean bout length was reported in 2 (29%) studies, ranging from 10.7 to 13.5 seconds [37,40]. Two studies (29%) reported daytime and nighttime PA, with David et al [31] reporting a mean of 168.1 (SD 30.05) activity counts during daytime hours versus a mean of 25.82 (SD 11.79) activity counts for nighttime hours and Lu et al [57] reporting a mean of 1593 (SD 44) CPM during waking hours and a mean of 245 (SD 9) CPM during sleeping hours. Mahlberg and Walther [51] also reported a mean of 35.2 (SD 20.5) activity counts per hour during nocturnal hours. van Alphen et al [33] described community dwellers with dementia to be the most active between 9 and 10 AM and between 2 and 3 PM, with significantly less physical activity than controls from 11 AM to 1 PM. The longest walking bout duration was reported as a mean range between 89.9 and 200.5 seconds [37].

### Variability

#### MCI Measurement

Of the 9 (27%) studies examining the variability of HPA, 2 (22%) characterized relevant metrics in MCI. Hayes et al [32] reported the variability of daily activity, with a mean coefficient of variance of 0.47 (SD 0.012), while Del Din et al [44] captured the variability of walking bout lengths (S^2^), with a mean range between 0.562 and 0.605.

#### Global Cognitive Impairment/Mixed Groups

We found 1 (3%) study that captured the variability of HPA in a mixed cognitive group (MCI and dementia). Similar to Del Din et al [44], Mc Ardle et al [38], reported a mean variability of walking bout length (S^2^) of 0.819 (SD 0.081).

#### Dementia

Two (6%) studies captured the variability of HPA in mild-moderate dementia [59,60]. Hooghiemstra et al [59] characterized intradaily variability, with a mean of 0.46 (SD 0.16) and interdaily variability, with a mean of 0.79 (SD 0.10). Meanwhile, Abel et al [60] reported day-to-day variability in terms of intracorrelation coefficients (ICCs) as >.70 for Friday to Sunday. They also reported ICCs for Friday to Saturday and Saturday to Sunday for hours spent walking, step count, and the number of walking bouts and highlighted the mean scores for each metric for each day (Multimedia Appendix 5) [60]. They reported significant differences between Friday, Saturday, and Sunday for volume characteristics *(P<.*001).

**Figure 3:**
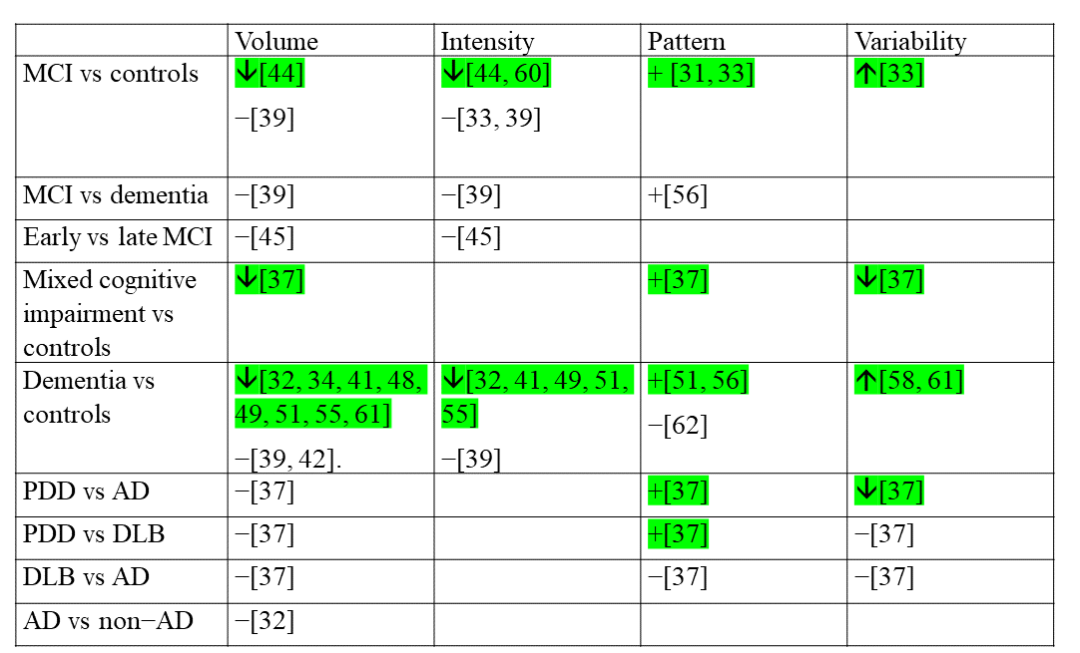
Significant differences in physical activity domains across the cognitive spectrum and between dementia disease subtypes Green indicates where significant differences have been reported. Where possible, directionality of results have been reported: arrows up indicates the former group have higher scores while arrows down indicates the former group have lower scores. Where directionality is not appropriate, we have marked significant differences with a + symbol. AD: Alzheimer disease; DLB: dementia with Lewy bodies; MCI: mild cognitive impairment; PDD: Parkinson disease dementia.

Results pertaining to significant differences in HPA across the cognitive spectrum and between dementia disease subtypes can be found in Figure 3.

## Discussion

### Principal Findings

**Figure 4:**
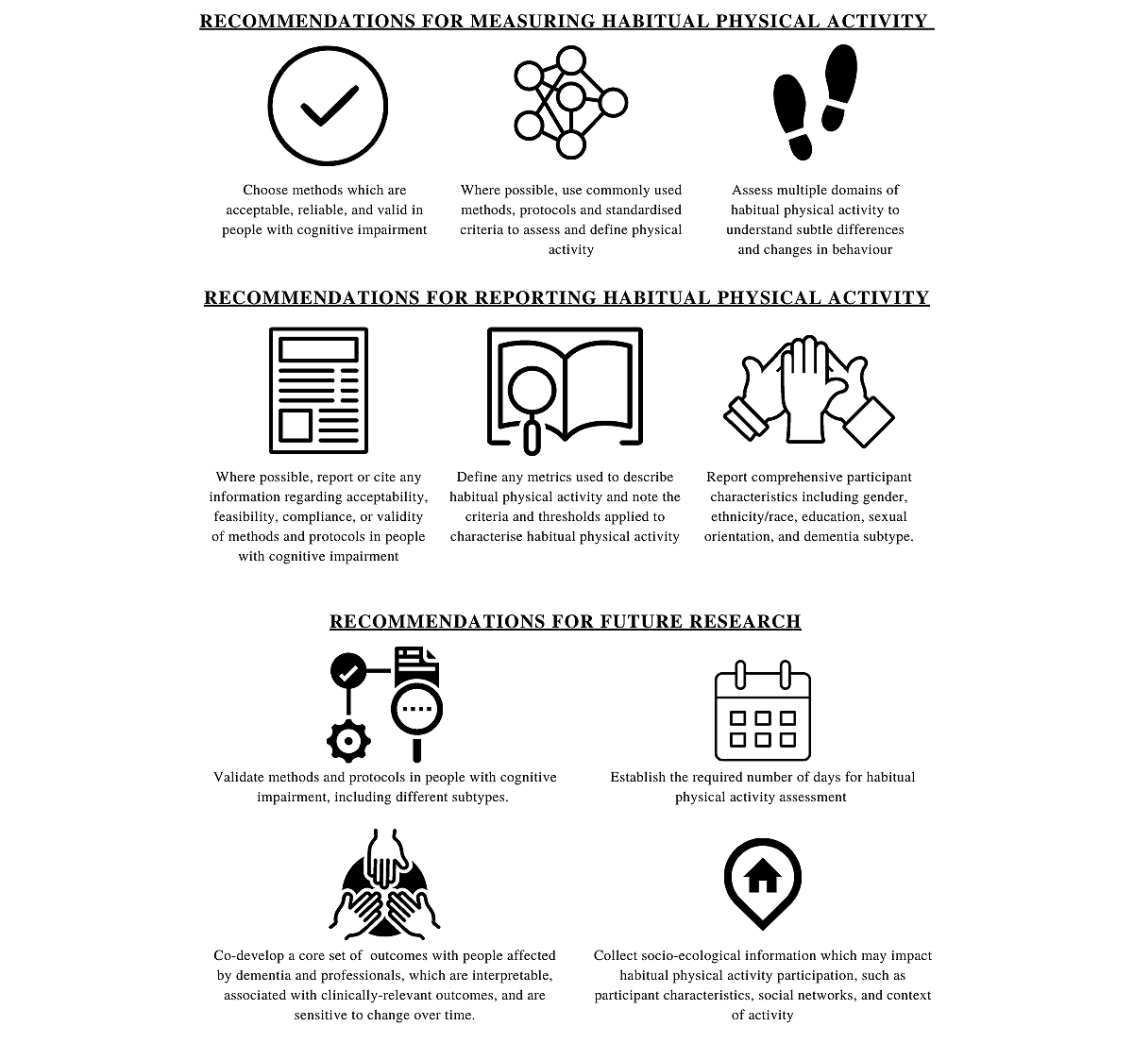
Key recommendations for assessing habitual physical activity in people with cognitive impairment.

This is the first systematic review to report the digital methods, protocols, and metrics used to capture HPA in people with cognitive impairment and to explore differences in HPA between levels of cognitive impairment and disease subtypes. Key findings highlighted that accelerometers worn on the wrist or lower back were the most prevalent method, while metrics relating to volume (eg, steps per day) were the most common characterization of HPA in this population. People with dementia are less physically active than those with normal cognitive function, showing lower volumes and intensity, with significant differences in variability metrics and daytime patterns of HPA. Findings in people with MCI varied, but they may have different patterns of HPA compared to those with normal cognition. We highlight key recommendations for measuring and reporting HPA and to guide future directions for research in this area, as summarized in Figure 4.

### Measuring HPA in People With Cognitive Impairment

Consistent with a recent review quantifying HPA in aged care, a variety of protocols, measurement devices, placement of devices, assessment periods, metrics, and criteria to characterize HPA (eg, cutoff thresholds) were used across the studies [19]. Limited information was provided regarding participant compliance and acceptability regarding these methods and protocols. All factors relating to methods and protocols can majorly impact results, and the interpretation of research findings is limited by the lack of standardization. For example, 3 (9%) studies reported daily step count as >10,000, significantly higher than most of the studies that recorded this metric [15,38,57]. Of these, 2 (66%) studies suggested that this was due to a lower cutoff threshold for quantifying HPA (ie, anything over 3 continuous steps) [15,38]. These findings highlight how the algorithm applied can alter results and emphasize the importance of validating the devices and methods used within a population with cognitive impairments. It should be noted that over half (n=17, 51%) of the studies included in this review did not report or cite information regarding the validity of the devices or methods used [31,33,35,39,40,42,43,46,48, 49,51,53,54,59,61-63]. Of those that did, devices and methods were validated in older adults [36,37,41,52,56,60], general adults [15,34,38,44,45,57,64], middle-aged females [50], people with Parkinson disease [37], and people with dementia [55]. Studies should cite or report validation results when possible to facilitate a greater understanding of discrepancies across the literature. Further work is required to ensure commonly used devices and methods are valid, acceptable, and accurate for people across the spectrum of cognitive impairment.

Of the 44 metrics captured across these studies, 28 (64%) were used only once (Table 2). Data synthesis is therefore difficult and reduces our understanding of how people with cognitive impairment participate in HPA. Metrics relating to volume (ie, steps, walk time, bouts, and activity counts per day) and intensity (ie, light physical activity or MVPA) were most commonly applied across multiple studies. When considering future studies, these metrics may be useful to employ for comparative purposes. Certain HPA metrics, such as root mean square difference, are not readily understandable and require an understanding of accelerometry, which may limit their use for clinical purposes. Similarly, a number of studies (n=7, 21%) linked global cognition and discrete cognitive domains (ie, information processing, executive function, visual perceptual abilities) with volume, intensity, and pattern of HPA [15,30,38,40,45,46,56], but there was a limited number exploring relationships between HPA and other clinically meaningful measures, such as functional independence and psychological or social outcomes. There were also no longitudinal observational studies identified for this review, which prevents minimal clinically meaningful differences and sensitivity to change measures to be quantified. Given that the loss of HPA has significant impacts on independence, health, socialization, and mental health [11,65] and is influenced by multiple socioecological factors [13], we suggest that a core set of interpretable HPA metrics should be identified which are associated with clinically important outcomes for people with cognitive impairment and their clinicians [66]. These should be monitored longitudinally to improve our understanding of trajectories of HPA change in people with cognitive impairment and how this change reflects the loss of independence, social networks, health, and psychological well-being.

Over half (n=17, 51%) of the studies measured HPA continuously for 7 days. However, protocols varied from 2 days to 3 months of continuous data collection within this review. In the general older adult population and in aged care, up to 5 days of data capture is considered sufficient for reliably estimating HPA outcomes using wearable devices worn on the trunk [67,68]. The most common continuous time period is generally 7 days, as it captures both weekdays and weekends, where structural differences are more likely to occur [60]. Key findings in this review indicate that the HPA of people with dementia varies between days, suggesting that they may have dynamic daily routines and habitual patterns, which may be affected by their degree of dependency [60,62]. Individuals with more severe cognitive impairment will have a greater reliance on social support, while those with less severe cognitive impairment may manage more of their daily activities independently, leading to structural differences in HPA (eg, between weekdays and weekends) [60]. This has important considerations for the interpretation of data and HPA assessment protocol, as averaging across days may overlook nuances in people’s behaviors [68]. There is a clear need for research to establish the required number of days for HPA assessment in people with cognitive impairment in the community to ensure that the information contributing to clinical decision-making is accurate and reliable.

### Differences In HPA Across the Cognitive Spectrum

Based on the studies included in this review, HPA is significantly different across all domains of measurement described in people with dementia, while the groups with MCI appeared to have different patterns of daytime activity and higher intraindividual and intradaily variability. This suggests that while the amount of HPA does not significantly change in the early stages of cognitive impairment, the way it is carried out is different. For example, patterns of daytime activity in HPA may reflect an individual’s routine, which may change over the course of the disease due to a range of socioecological factors [13]. Time-series analysis may be useful to capture the loss of functional independence; for example, a person with cognitive impairment may show a decrease in peak activity times if they are no longer responsible for key household tasks, such as cleaning or grocery shopping [38,64]. As previously highlighted in this paper, establishing associations between different HPA metrics and clinically or functionally relevant outcomes may provide new insights into the hierarchy of HPA loss across the cognitive spectrum.

### Limitations and Suggestions for Future Research

Key limitations and suggestions for future research have already been highlighted regarding the lack of validity and consistency in methods and metrics reported, the lack of longitudinal research, and the limited associations between HPA metrics and clinically meaningful outcomes. A further limitation is the lack of information regarding the representativeness and inclusivity of the participant samples included in the studies in this review. Only 3 (9%) studies reported ethnicity [45,47,52], and 2 (6%) compared HPA in different dementia subtypes [33,38]. Moreover, only 1 (3%) study recruited early onset dementia, limiting our understanding of HPA in people with younger onset cognitive impairment [59]. It is unclear whether discrete dementia disease stages (ie, MCI vs dementia, mild vs moderate dementia) participate differently in HPA due to the limited research [69]. It is important to ensure the recruitment of participants from underserved groups, such as ethnic minorities, lower socioeconomic status, rarer or more advanced dementias, or people living in remote areas, so that results are generalizable and inform the development of inclusive interventions for health and social care. We need to understand physical activity participation in underserved groups like these to ensure that we are creating inclusive support strategies and interventions. Feasibility studies should be prioritized within these groups to ensure protocols, devices, and metrics used are acceptable and usable across the general population with cognitive impairments.

### Strengths and Limitations

This systematic review had several strengths, including a comprehensive search strategy and the use of multiple databases used to screen potential articles for inclusion. Independent title, abstract, and full-text screening was carried out, with a third reviewer adjudicating disagreements. Our quality assessment suggested that most studies were moderate-to-good quality. However, based on our resources, this review was limited due to the inclusion of only articles written in English, which may have led to the exclusion of relevant studies published in other languages. Additionally, we only included HPA metrics that captured ambulatory activities, excluding functional HPA characteristics, such as standing or sitting time. These were beyond the scope of our review, but understanding how people with cognitive impairment participate in these should also be considered, as changes in ambulatory HPA will impact functional HPA (ie, more ambulatory movement may lead to less time sitting or standing) [70]. Because this is a systematic review, we were unable to account for confounding variables that may impact physical activity in people with cognitive impairment, such as multimorbidity or poor physical health; however, this should be acknowledged in future research studies. Due to the huge range of metrics, protocols, and criteria to characterize HPA, a meta-analysis was not an appropriate way to synthesize these data but should be considered as the field develops and standardizes.

### Conclusion

Wearable technology (eg, accelerometers) is the most common HPA assessment tool, while metrics relating to volume are the most prevalent to describe HPA in people with cognitive impairment. People with dementia have lower volumes and intensities of HPA compared to normal aging, and people with both MCI and dementia show different patterns and higher variability of HPA compared to controls. The lack of standardization across methods and metrics limits our understanding; therefore, more inclusive recruitment strategies are needed in future studies to establish how people with cognitive impairment participate in HPA. Future research needs to consider longitudinal studies to understand HPA change in people with cognitive impairment and how this reflects clinically relevant outcomes.

## Appendix

Multimedia Appendix 1 Search strategies employed in this systematic review.

Multimedia Appendix 2 PRISMA (Preferred Reporting Items for Systematic Reviews and Meta-analyses) checklist.

Multimedia Appendix 3 Key demographics and physical activity assessment tools of included studies.

Multimedia Appendix 4 Quality assessment of all studies included in this systematic review.

Multimedia Appendix 5 Key results relating to habitual physical activity across all studies included in this review.

## Abbreviations

CPM: counts per minute
HPA: habitual physical activity
ICC: intracorrelation coefficient
MCI: mild cognitive impairment
MET: metabolic equivalent
MVPA: moderate-vigorous physical activity
PRISMA: Preferred Reporting Items for Systematic Reviews and Meta-analyses
WHO: World Health Organization
