# Supplementary material for: Using Digital Technology to Quantify Habitual Physical Activity in Community Dwellers With Cognitive Impairment: Systematic Review
Source: J Med Internet Res. 2023 May 18;25:e44352. doi: 10.2196/44352 (PMC10236281; doi:10.2196/44352)
Supplement: Multimedia Appendix 1 [file jmir_v25i1e44352_app1.docx]

**Appendix 1: Search Strategy**

*Original String:*

| ***Cognitive impairment*** | (dementia* OR "cognitive* impair*" OR "cognitive* dysfunction" OR Alzheimer* OR vascular OR Lewy*) |
| --- | --- |
| ***Physical activity*** | ("physical activit*" OR movement OR step OR walk* OR ambulation* OR exercise* OR "motor activit*" OR mobility* OR sedentary) |
| ***Digital technology*** | (acceleromet* OR "fitness tracker*" OR sensor OR actigraph* OR wearable* OR smartwatch* OR "Smart watch" OR "activity track*") |

***Search via Scopus***

( TITLE-ABS-KEY ( ( dementia* OR "cognitive* impair*" OR "cognitive* dysfunction" OR alzheimer* OR vascular OR lewy* ) ) AND TITLE-ABS-KEY ( ( "physical activit*" OR movement OR step OR walk* OR ambulation* OR exercise* OR "motor activit*" OR mobility* OR sedentary ) ) AND TITLE-ABS-KEY ( ( acceleromet* OR "fitness tracker*" OR sensor OR actigraph* OR wearable* OR smartwatch* OR "Smart watch" OR "activity track*" ) ) )

**Reran November 2021**

( TITLE-ABS-KEY ( ( dementia* OR "cognitive* impair*" OR "cognitive* dysfunction" OR alzheimer* OR vascular OR lewy* ) ) AND TITLE-ABS-KEY ( ( "physical activit*" OR movement OR step OR walk* OR ambulation* OR exercise* OR "motor activit*" OR mobility* OR sedentary ) ) AND TITLE-ABS-KEY ( ( acceleromet* OR "fitness tracker*" OR sensor OR actigraph* OR wearable* OR smartwatch* OR "Smart watch" OR "activity track*" ) ) ) AND ( LIMIT-TO ( PUBYEAR , 2021 ) OR LIMIT-TO ( PUBYEAR , 2020 ) )

********

***Search via Web of Science***

(dementia* OR "cognitive* impair*" OR "cognitive* dysfunction" OR Alzheimer* OR vascular OR Lewy*) (All Fields) and ("physical activit*" OR movement OR step OR walk* OR ambulation* OR exercise* OR "motor activit*" OR mobility* OR sedentary) (All Fields) and (acceleromet* OR "fitness tracker*" OR sensor OR actigraph* OR wearable* OR smartwatch* OR "Smart watch" OR "activity track*") (All Fields) and Article (Document Types) and English (Languages)

**Reran November 2021:**

(dementia* OR "cognitive* impair*" OR "cognitive* dysfunction" OR Alzheimer* OR vascular OR Lewy*) (All Fields) and ("physical activit*" OR movement OR step OR walk* OR ambulation* OR exercise* OR "motor activit*" OR mobility* OR sedentary) (All Fields) and (acceleromet* OR "fitness tracker*" OR sensor OR actigraph* OR wearable* OR smartwatch* OR "Smart watch" OR "activity track*") (All Fields) and Article (Document Types) and 2020 or 2021 or 2022 (Publication Years) and English (Languages)

****

***Search ran via Ovid across multiple databases, including:***

Ovid MEDLINE(R) <1946 to current>

Embase <1974 to current>

APA PsycArticles Full Text

APA PsycInfo <1806 to current>

1 ((dementia* or "cognitive* impair*" or "cognitive* dysfunction" or Alzheimer* or vascular or Lewy*) and ("physical activit*" or movement or step or walk* or ambulation* or exercise* or "motor activit*" or mobility* or sedentary) and (acceleromet* or "fitness tracker*" or sensor or actigraph* or wearable* or smartwatch* or "Smart watch" or "activity track*")).mp.

2 limit 1 to english language [Limit not valid in Journals@Ovid; records were retained

3 limit 2 to human [Limit not valid in Journals@Ovid; records were retained]

4 limit 3 to humans [Limit not valid in Journals@Ovid,APA PsycInfo; records were retained]

5 limit 4 to original articles [Limit not valid in Ovid MEDLINE(R),Embase,APA PsycInfo; records were retained]

6 remove duplicates from 5

**Reran November 2021, with added limit:**

7. limit 6 to yr="2020 - 2021"
