# Supplementary material for: Using Digital Technology to Quantify Habitual Physical Activity in Community Dwellers With Cognitive Impairment: Systematic Review
Source: J Med Internet Res. 2023 May 18;25:e44352. doi: 10.2196/44352 (PMC10236281; doi:10.2196/44352)
Supplement: Multimedia Appendix 3 [file jmir_v25i1e44352_app3.docx]

Appendix 3: Key demographics and physical activity assessment tools of included studies

| **Study** | **Participant Demographics** | **Diagnostic criteria** | **Country** | **Study design** | **Physical activity assessment tool** |
| --- | --- | --- | --- | --- | --- |
| Abel [60] | 53 mild-moderate dementia Age: 82.3±5.6 years 73.6% female Education: 11.8±3.4 years MMSE: 22.1±2.9 Max gait speed: 1.05±0.37 m/s TUG: 17±7.2 seconds | All cause dementia: McKhann 2011 | Germany | Baseline results from RCT | Accelerometer: PAMSys, Biosenics. Worn via chest strap on sternum for 3 days. |
| Bongartz [30] | 110 mild-moderate dementia (94 complete datasets). Age: 82.3±5.9 Sex: not specified.  MMSE: 23.3±2.4 Gait Speed: 0.46±0.19 m/s TUG: 24.4±14.1 | Dementia, unspecified criteria | Germany | Baseline results from RCT | IMU: uSense, worn on lower back for 48 hours. Accelerometer: PAMsys, Biosensics, worn via chest strap on sternum for 48 hours. |
| Buckley [64] | 36 mild Alzheimer's disease Age: 77±6 58% female MMSE: 23±4  26 controls Age: 74±9 58% female MMSE: 29±1 | AD: McKhann 2011 | UK | Cross-sectional | Accelerometer: Axivity AX3, worn on the lower back for 7 days, 24 hours a day. |
| Cerff [40] | 17 PD-NC: Age: 71 (44-80) 41% female. UPDRS-III: 20 (11-58)  22 PD-MCI: 68 (57-78) 23% female UPDRS-III: 24 (10-62)  9 PDD Age: 72 (67-75) 0% female UPDRS-III: 36 (14-56) | PD-NC PD-MCI, 1 or more test scores ≥ 1.5 SDs below published normative values for healthy normal controls in cognitive domains; no significant ADL impairment, no primary explanations for cognitive impairment/PD-associated symptoms which affect cognitive testing. PDD: Emre 2007 | Germany | Pilot study | Accelerometer: DynaPort Minimod (Mc Roberts), worn on the lower back for 3 days |
| Chang [46] | 54 MCI Age: 69.7±8 57% female Education: 8.7±5 years.  CDR: 0.5 | Amnestic-MCI. Global CDR score of 0.5 and ≥0.5 for memory domain. Memory loss scores 1.5 SD below the education-adjusted normative mean.  No dementia. | Taiwan | Cross-sectional | Accelerometer, Xiami Mi Band devices (GPS-enabled), worn on the wrist for 7 days (data removed first and last day). |
| David [31] | 107 AD Age: 77.2±6.69 54% female MMSE: 21.4±5.31 | AD: McKhann 1984 | France/USA | Cross-sectional | Actigraph, Micromini motion logger worn on the non-dominant wrist for 7 days |
| Del Din [44] | 38 fallers with MCI Age: 78.03±6.21 68% female MMSE: 25.95±2.4  109 Older adult fallers Age: 75.93±6.22 78% female MMSE: 28.52±1.36  128 fallers with PD Age: 71.63±6.43 37% female MMSE: 28.07±1.68 | MCI: Score of 0.5 on CDR | UK, Belgium, the Netherlands, Israel, Italy | Intervention, baseline data | Accelerometer, Axivity AX3, worn on lower back for 7 days |
| Doi [53] | 263 MCI with non-severe WML Age: 70.7±4.1 Sex: 54.7% female a-MCI: 51.8% na-MCI: 48.2% TUG: 8.4±1.7 secs  60 MCI with severe WML Age: 74.3±5.2 Sex: 50% female a-MCI: 45.2% na-MCI: 54.8% TUG: 9±1.7 secs | MCI: Petersen, 2004 | Japan | Cross-sectional | Accelerometer, modified HJA-350IY, Active Style Pro, Omron Healthcare Co., worn on hip for 2 weeks. |
| Eckert [63] | 63 mild-moderate dementia Age: 82.2±5.8 76.2% female Low education: 33% Middle education: 47.6 High education: 19% MMSE: 23.3±2.7 SPPB:5.4±2.1 | Mild-moderate dementia, criteria not described | Germany | Baseline results from RCT feasibility | Accelerometer, PAMsys, Biosensics worn for 48 hours, placement not specified |
| Falck [45] | 69 Controls MMSE: 29.22±.87 MoCA: 27.19±1.1  81 MCI MMSE: 28.65±1.15 MoCA: 11.84±2.11  Overall:  Age: 71.11±7.22 67.10% 18.4% High school diploma or less. 11.20% Trade School  15.8% Some University 54.6% University diploma or higher. | Probable MCI, <26 on MoCA | Canada | Cross-sectional | Accelerometer, MotionWatch 8 worn on non-dominant wrist for ≥4 days |
| Finnanger [55] | 29 dementia in farm-based care Age: 74±7.22 31% female Primary school education: 13.8% Secondary school education: 65.5% University education: 20.7% TUG: 13.48±5.61 seconds CDR: 1.22±0.57  107 dementia in day care.  Age: 84.3**±**8.1 years 66% female Primary school education: 46% Secondary school education: 23% University education: 28.7% CDR: 1.53±0.67 | Not specified | Norway | Mix of two studies:  Longitudinal - time point 2, cross sectional | Actigraph: Actisleep+, Actigraph, worn on left wrist for 7 days. Must have 3 valid days with 8+ hours recorded |
| Harada [39] | 192 CI Age: 76.2±4.1 Sex: 46% female Education: 12±2.6 years 6-minute walk: 452.6±55.3 metres | Global cognitive impairment, 21-24 on the MMSE. | Japan | Baseline results from RCT | Accelerometer: GT40-20, ACOS Corporation, Nagano, Japan worn on waist.   GPS monitor: Globalsat DG-200 Data Logger, Global Sat WorldCom, Taipei, Taiwan, worn on waist.  Wear time: 14 days - included with at least 8 days with 10+ hours wear time. |
| Harada [48] | 262 CI, Age: 76.2±4.1 44.1% female Education: 11.9±2.6 years | Global cognitive impairment, 21-24 on the MMSE. | Japan | Baseline results from RCT | Accelerometer: GT40-20, ACOS Corporation, Nagano, Japan worn on waist for 14 days |
| Hartman [50] | 45 Dementia Age: 79.6±5.9 48.9% female MMSE: 22.8±3.2 55.5% AD 4.4% VaD 26.7 mixed dementia 13.3% not specified dementia  49 Controls Age: 80±7.7 51% female MMSE: 29±1.2 | Dementia, as assessed via comprehensive assessment with a clinician | Not specified | Cross-sectional | Accelerometer, Philips Activwatch 2 worn on non-dominant wrist for 7 days, 24 hours a day. |
| Hausdorff [61] | 100 controls Age: 76±6.2 78% female Education: 13.4±3.9 years MoCA: 25.8±2.6 Gait Speed: 1.06±0.24 m/s  36 MCI:  Age: 77.8±6.4 72% female Education: 10.9±2.9 MCI: 21.3±4.1 Gait Speed: 0.90±0.29 m/s | MCI, based on 0.5 on CDR | Belgium, Israel, Italy, the Netherlands, the UK | Baseline results from RCT | Accelerometer, Axivity AX3, worn on lower back for 7 days |
| Hayes [32] | 7 Controls 71% female  MMSE: 27-29 ADLS: 0-1 UPDRS-III: 0-7  7 MCI 57% female MMSE: 25-28 ADLS: 0-2 UPDRS-III: 1-5  Overall:  Age: 89.3±3.7 Education: 12-20 years | MCI, based on 0.5 on CDR | USA | Cross-sectional | Passive infrared pyroelectric motion sensors (MS16A, x10.com) placed in every room of the house at locations expected to pick up movement, measured for 26 continuous weeks |
| Hooghiemstra [59] | 61 mild-moderate early onset dementia Age: 63.5 (50-71) 33% female Low education: 6.6% Medium education: 41% High education: 52.5% MMSE: 25 (16-30) AD: 75.4% VaD: 8.2% DLB: 11.5% FTD: 3.3% Unspecified dementia: 1.6%  68 controls Age: 63 (54-69) 59% female Low education: 2.9% Medium education: 49.4% High education: 47.8% MMSE: 30 (26-30) | Dementia: DSM V  AD: McKhann 2011 FTD: Neary 1998 DLB: McKeith 2005 | The Netherlands | Cross-sectional | Piezoelectric sensor: Actiwatch 4 worn on dominant wrist for 7 days. |
| Iwata [42] | 14 dementia Male age: 74.2 Female age: 75.4 64% female  Male MMSE: 16.8 Female MMSE: 19.9 Male FAQ: 21.2 Female FAQ: 15.7   16 controls (including 10 MCI) Male age: 72.7 Female age: 74.6 31% female  Male MMSE: 27.6 Female MMSE: 26 Male FAQ: 0.5 Female FAQ: 1 | Dementia: DSM IV Probable AD: McKhann 1984 MCI: Petersen 2004 | Japan | Cross-sectional | Accelerometer: HJA-350IT-OMRON, unspecified placement, worn for 2-3 months during waking hours. |
| James [35] | 694 participants 10.1% with clinical dementia Age: 82.2±7 75.9% female 93.7% white Education: 14.7±3 years MMSE: 27.3±3.4 Katz index: 0.2±0.7 | Dementia, based on clinician diagnosis | USA | Cross-sectional | Actigraph: Actical, Mini Mitter, worn for 10 days, 24 hours a day, on the non-dominant wrist. |
| Kwan [41] | 33 MCI Age: 71 (IQR: 9) 85% female 52% completed secondary school or above. MoCA: 21 (IQR: 6.5) | MCI: Albert 2011 | Hong Kong | Baseline RCT results | Accelerometer: Actigraph GT3Xm worn on wrist for 7 days, 24 hours a day, included if worn for 10>hours for 3> days. |
| Lu [57] | 271 controls Age: 81.9±3.5 38.3% female Education: 8.7±4.9 years MoCA: 24.4±2.4 Gait speed (m/s): 0.88±.21  252 low MoCA score. Age: 83.4±4 47.6% female Education: 6.3±4.8 years  MoCA: 19.1±3.1 Gait speed (m/s): 0.78±.21  105 a-MCI Age: 83.6±3.7 48.6% female Education: 4.5±4.4 years MoCA: 18.1±3 Gait Speed (m/s): 0.77±.19  182 AD Age: 80.8±5.9 65.4% female Education: 5.3±4.8 years MoCA: 13.1±5.1  Gait Speed (m/s): 0.71±.21 | AD: McKhann 1984 a-MCI: clinical diagnosis from two experienced clinicians | Hong Kong | Cross-sectional analysis at follow up longitudinal visit | Accelerometer: Actigraph GT3Xm worn on wrist for 7 days, 24 hours a day, included if worn for 10>hours for 3> days. |
| Mahlberg and Walther [51] | 24 AD, split into intervention and placebo.  14 Intervention AD Age: 79±9 57% female  10 Placebo AD Age: 78.2±10.3 60% female  10 older controls Age: 84.3±4.2 50% female  10 younger controls Age: 22.6±1 50% female | AD: McKhann 1984 | Germany | Baseline results from drug trial intervention | Accelerometer: Actiwatch worn on non-dominant wrist for two days. |
| Mc Ardle [15] | 16 mild AD Age: 70.9±8.3 50% female MMSE: 25±3 BADLS: 3±3 | AD: McKhann 2011 | UK | Feasibility study | Accelerometer, Axivity AX3, worn on lower back for 7 days |
| Mc Ardle [38] | 36 AD Age: 77±6 58% female MMSE: 23 (14-29) BADLS: 6 (0-31) UPDRS-III: 7 (0-19)  30 DLB Age: 76±6 20% female MMSE: 24 (16-30) BADLS: 13 (3-24) UPDRS-III: 26 (5-57)  16 PDD Age: 79±6 13% female MMSE: 24 (12-30) BADLS: 11 (1-31) UPDRS-III: 41 (20-78)  26 Controls Age: 74±9 58% female MMSE: 30 (25-30) BADLS: 0 (0-1) UPDRS-III: 1 (0-11) | AD: McKhann 2011 DLB: McKeith 2017 PDD: Dubois 2007  AD-MCI: Albert 2011 PD-MCI: Litvan 2012 DLB-MCI: McKeith 2020 | UK | Cross-sectional | Accelerometer, Axivity AX3, worn on lower back for 7 days |
| Nickerson and Shade [47] | 14 MCI Age: 76.21±2.69 (72-81) 50% female 85.7% European American 7.1% African American 7.1% Asian MoCA: 22.14±2.57 (18-25) TUG: 9.43±1.26 (6-11) | Not specified | USA | Observational | Activity tracker: Withings Activite, worn on wrist for 12 weeks |
| Pedroso [49] | 30 Controls Age: 74.1±5.6 Sex not described. Education: 4.5±3.7 years MMSE: 24.6±4 DAFS: 85.5 (73-97) TUG (secs): 7.3±1.8  24 AD Age: 76.9±5.3 MMSE: 19.8±4.5 DAFS: 62.5±55-72 TUG (secs): 8.6±1.9 | Probable AD: DSM IV | Brazil | Cross-sectional | Pedometer, Yamax Digiwalk pedometer, worn for 7 days, unspecified placement. |
| Rackoll [34] | 18 MCI Age: 70±8 56% female Education: 16±5 years MMSE: 28±1  48 controls Age: 65±6 52% female Education: 16±3 years MMSE: 29±1 | amnestic MCI or amnestic MCI plus: Petersen 2004 | Germany | Intervention, baseline data | Actigraph: Actigraph GT3X+ worn on hip for 7 days |
| Rawtaer [43] | 21 Controls Age: 73±5.3 67% female Education: 7±4 years  28 MCI Age: 75.1±6.3 68% female Education: 4.53.9 years | MCI: Petersen 2004 | Singapore | Cross-sectional | Wearable activity band, Microsoft band, worn on wrist for 2 months. |
| Schwenk [37] | 28 Fallers with Dementia Age: 82±7.1 years 88% female MMSE: 22±3.4 Barthel Index: 81.6±16.4 TUG: 13.3±5.9 secs  49 non-fallers with dementia Age: 81.8±5.9 years 65% female MMSE: 22.1±3.1 Barthel Index: 83.2±12.9 TUG: 14.3±5.4 | AD: McKhann 1984 VaD: Roman 1993 | Germany | Cross-sectional | IMU: Physilog worn via chest strap on sternum for 24 hours. |
| Taylor [56] | 45 mild-moderate dementia Age: 81.4±6.4 42% female Education: 11 years (9-15) MoCA: 16 (11-19) TUG: 12.1 secs (11.2-16.3) Gait speed: 0.77±0.24 m/s  90 controls Age: 81.2±5.8 42% female Education: 15 years (12-18) MoCA: 26 (24-27) TUG: 8.2 secs (7-9.8) Gait speed: 1.20±0.24m/s | Specialist clinical diagnosis, MMSE<24, ACE-III <83 | Australia | RCT, baseline | Accelerometer: DynaPort MoveMonitor, McRoberts, worn on an elastic belt around trunk (Lumbar spine region) for 7 days - included if worn for at least 2 days, 6 hours each. |
| van Alphen [33] | 37 community-dwellers with dementia Age: 77.3±5.6 years 40.5% female MMSE: 20.8±4.8  Alzheimer's disease: 48.6% Vascular dementia: 16.2% Alzheimer's with vascular problems: 10.8% Dementia with Lewy bodies: 8.1% Frontotemporal dementia: 2.7% Parkinson's disease dementia: 5.4% Korsakoff dementia: 2.7% Unspecified dementia: 5.4%  83 nursing home residents Age: 83±7.6 79.5% female MMSE: 15.5±6.5  Alzheimer's disease: 49.4% Vascular dementia: 14.5% Alzheimer's with vascular problems: 9.6% Dementia with Lewy bodies: 0% Frontotemporal dementia: 6% Parkinson's disease dementia: 0% Korsakoff dementia: 2.4% Unspecified dementia: 18.1%  26 controls Age: 79.5±5.6 years 50% female MMSE: 28.2±1.6 | Not specified | The Netherlands | Cross-sectional | Accelerometer: Actiwatch (AW-4) worn on dominant wrist for <6 days, 24 hours per day. |
| Varma and Watts [52] | 39 Alzheimer's disease Age: 73.5±7.9 years 28.2% female 87.2% white 10.5% with a mobility impairment  53 controls Age: 73.2±6.5 years 69.8% female 96.2% white 11.3% with a mobility impairment | AD: McKhann 1984 | USA | Cross-sectional | Accelerometer: Actigraph GT3X+ worn on the hip for 7 days, 24 hours a day. |
| Watts [62] | 33 mild Alzheimer's disease Age: 72.73±7.47 years 30.3% female Education: 15.61±  53 controls Age: 73.2±6.5 years 69.8% female Education: 17.32±3.38 years | AD: McKhann 1984 | USA | Cross-sectional | Accelerometer: Actigraph GT3X+ worn on the hip for 7 days, 24 hours a day. |

Abbreviations: MMSE = Mini mental state examination, TUG = Timed up and Go test, RCT = randomised control trial, UK = United Kingdom, PD = Parkinson’s disease, NC = normal cognition, MCI = mild cognitive impairment, PDD = Parkinson’s disease dementia, SD = standard deviation, ADL = activities of daily living, UPDRS-III = Unified Parkinson’s disease Rating Scale III, CDR = clinical dementia rating, AD = Alzheimer’s disease, USA = United States of America, WML = white matter lesions, a-MCI = amnestic MCI, na-MCI = non-amnestic MCI, SPPB = Short physical performance battery, MoCA = Montreal Cognitive assessment, VaD = vascular dementia, DLB = dementia with Lewy bodies, FTD = frontotemporal dementia, BADLS = Bristol activities of daily living scale, DAFS = Direct assessment of functional status.
