# Supplementary material for: Using Digital Technology to Quantify Habitual Physical Activity in Community Dwellers With Cognitive Impairment: Systematic Review
Source: J Med Internet Res. 2023 May 18;25:e44352. doi: 10.2196/44352 (PMC10236281; doi:10.2196/44352)
Supplement: Multimedia Appendix 4 [file jmir_v25i1e44352_app4.docx]

Appendix 4: Quality assessment of all studies included in this systematic review.

|  |  | **Was the research question/objective in this paper clearly stated?** | **Was the study population clearly specified and defined?** | **Were withdrawals reported and explained?** | **Were inclusion/exclusion criteria for participants defined and determined prior to study onset?** | **Was a sample size justification, power description or variance and effect estimates provided?** | **Were the outcome measures (dependent variables) clearly defined, valid, reliable, and implemented consistently across all study participants?** | **Were clinical diagnostic criteria and severity ratings for dementia reported and adhered to?** | **Were key potential confounding variables measured and adjusted statistically for their impact on the outcome?** | **Scores** | **Average Score** |
| --- | --- | --- | --- | --- | --- | --- | --- | --- | --- | --- | --- |
| Abel et al., 2019 | *R1* | Y | N | N | Y | N | Y | Y | Y | 5 | 5.5 |
|  | *R2* | Y | Y | N | Y | N | Y | Y | Y | 0 |  |
| Bongartz et al., 2019 | *R1* | Y | N | Y | Y | N | Y | N | N | 5 | 5 |
|  | *R2* | Y | Y | Y | Y | N | Y | N | N | 0 |  |
| Buckley et al., 2016 | *R1* | Y | N | N | N | N | N | Y | N | 2 | 2 |
|  | *R2* | Y | N | N | N | N | N | Y | N | 0 |  |
| Cerff et al., 2017 | *R1* | Y | Y | Y | Y | N | Y | Y | Y | 7 | 7 |
|  | *R2* | Y | Y | Y | Y | N | Y | Y | Y | 0 |  |
| Chang et al., 2017 | *R1* | Y | N | N | Y | N | Y | Y | Y | 5 | 5.5 |
|  | *R2* | Y | Y | N | Y | N | Y | Y | Y | 0 |  |
| David et al., 2012 | *R1* | Y | Y | Y | Y | N | N | Y | N | 5 | 5.5 |
|  | *R2* | Y | Y | Y | Y | N | Y | Y | N | 0 |  |
| Del Din et al., 2020 | *R1* | Y | N | N | Y | Y | Y | N | Y | 5 | 4.5 |
|  | *R2* | Y | N | N | Y | N | Y | N | Y | 0 |  |
| Doi et al., 2015 | *R1* | Y | Y | Y | Y | N | N | Y | Y | 6 | 6.5 |
|  | *R2* | Y | Y | Y | Y | N | Y | Y | Y | 0 |  |
| Eckert et al., 2020 | *R1* | Y | N | Y | Y | N | Y | N | N | 4 | 5 |
|  | *R2* | Y | Y | Y | Y | N | Y | N | Y | 0 |  |
| Falck et al., 2017 | *R1* | Y | N | N | Y | N | Y | N | Y | 4 | 4.5 |
|  | *R2* | Y | Y | N | Y | N | Y | N | Y | 0 |  |
| Finnanger et al., 2020 | *R1* | Y | N | Y | Y | N | Y | N | Y | 5 | 5 |
|  | *R2* | Y | N | Y | Y | N | Y | N | Y | 0 |  |
| Harada et al., 2016 | *R1* | Y | N | Y | N | N | Y | N | Y | 4 | 4.5 |
|  | *R2* | Y | N | Y | Y | N | Y | N | Y | 0 |  |
| Harada et al., 2019 | *R1* | Y | N | Y | N | N | Y | N | Y | 4 | 4.5 |
|  | *R2* | Y | N | Y | Y | N | Y | N | Y | 0 |  |
| Hartman et al., 2018 | *R1* | Y | Y | Y | Y | N | Y | N | Y | 6 | 6 |
|  | *R2* | Y | Y | Y | Y | N | Y | N | Y | 0 |  |
| Hausdorff et al., 2018 | *R1* | Y | Y | Y | Y | N | Y | N | N | 5 | 5 |
|  | *R2* | Y | Y | Y | Y | N | Y | N | N | 0 |  |
| Hayes et al., 2008 | *R1* | Y | N | Y | Y | N | N | N | N | 3 | 4.5 |
|  | *R2* | Y | Y | Y | Y | N | Y | Y | N | 0 |  |
| Hooghiemstra et al., 2015 | *R1* | Y | Y | N | Y | N | Y | Y | N | 5 | 5 |
|  | *R2* | Y | Y | N | Y | N | Y | Y | N | 0 |  |
| Iwata et al., 2013 | *R1* | N | N | N | Y | N | N | Y | N | 2 | 3 |
|  | *R2* | N | N | N | N | N | N | Y | Y | 0 |  |
| James et al., 2012 | *R1* | Y | N | Y | N | N | Y | N | N | 3 | 3 |
|  | *R2* | Y | N | Y | N | N | Y | N | N | 0 |  |
| Kwan et al., 2020 | *R1* | Y | Y | Y | Y | Y | Y | Y | N | 7 | 4.5 |
|  | *R2* | Y | Y | Y | Y | Y | Y | Y | Y | 0 |  |
| Lu et al., 2018 | *R1* | Y | Y | Y | N | N | Y | Y | Y | 6 | 6 |
|  | *R2* | Y | Y | Y | N | N | Y | Y | Y | 0 |  |
| Mahlberg et al., 2007 | *R1* | Y | N | N | Y | N | N | Y | N | 3 | 3.5 |
|  | *R2* | Y | Y | N | Y | N | N | Y | N | 0 |  |
| Mc Ardle et al., 2019 | *R1* | Y | Y | Y | Y | N | Y | Y | Y | 7 | 7 |
|  | *R2* | Y | Y | Y | Y | N | Y | Y | Y | 0 |  |
| Mc Ardle et al., 2018 | *R1* | Y | Y | Y | Y | N | Y | Y | N | 6 | 6.5 |
|  | *R2* | Y | Y | Y | Y | N | Y | Y | Y | 0 |  |
| Nickerson et al., 2020 | *R1* | Y | N | Y | Y | N | N | N | N | 3 | 4 |
|  | *R2* | Y | Y | Y | Y | N | N | N | Y | 0 |  |
| Pedroso et al., 2017 | *R1* | Y | N | N | Y | N | N | Y | N | 3 | 3 |
|  | *R2* | Y | N | N | Y | N | N | Y | N | 0 |  |
| Rackoll et al., 2021 | *R1* | N | N | N | Y | N | Y | Y | Y | 4 | 4 |
|  | *R2* | Y | N | N | Y | N | Y | N | Y | 0 |  |
| Rawtear et al., 2017 | *R1* | Y | N | Y | Y | Y | N | Y | N | 5 | 5 |
|  | *R2* | Y | N | Y | Y | Y | N | Y | N | 0 |  |
| Schwenk et al., 2014 | *R1* | Y | Y | N | Y | N | Y | Y | N | 5 | 5.5 |
|  | *R2* | Y | Y | N | Y | N | Y | Y | Y | 0 |  |
| Van alphen et al., 2016 | *R1* | Y | Y | Y | N | N | Y | N | Y | 5 | 5.5 |
|  | *R2* | Y | Y | Y | Y | N | Y | N | Y | 0 |  |
| Taylor et al., 2019 | *R1* | Y | Y | N | Y | N | Y | N | N | 4 | 4.5 |
|  | *R2* | Y | Y | N | Y | N | Y | N | Y | 0 |  |
| Varma et al., 2017 | *R1* | Y | Y | N | Y | N | Y | Y | Y | 6 | 6 |
|  | *R2* | Y | Y | N | Y | N | Y | Y | Y | 0 |  |
| Watts et al., 2016 | *R1* | Y | Y | Y | Y | N | Y | Y | Y | 7 | 7 |
|  | *R2* | Y | Y | Y | Y | N | Y | Y | Y | 0 |  |

Abbreviations: R1 = reviewer 1, R2 = reviewer 2, Y = yes, N = no.
