# Supplementary material for: Using Digital Technology to Quantify Habitual Physical Activity in Community Dwellers With Cognitive Impairment: Systematic Review
Source: J Med Internet Res. 2023 May 18;25:e44352. doi: 10.2196/44352 (PMC10236281; doi:10.2196/44352)
Supplement: Multimedia Appendix 5 [file jmir_v25i1e44352_app5.docx]

Appendix 5: Key results relating to habitual physical activity across all studies included in this review.

| **Study** | **Cognitive status** | **Main Study Findings** | **Comparisons** |
| --- | --- | --- | --- |
| Abel [60] | Mild-moderate dementia | **Variability:** Day-to-day variability (ICCs): >.70  Walking time hours Friday-Saturday (ICC): 0.28 (-.07-.57) Walking time hours Saturday-Sunday (ICC): .62 (.39-.78)  Walking episodes (number): Friday-Saturday (ICC): .16 (-.09, .42) Saturday-Sunday (ICC): .71 (.51,.84)  Steps (number): Friday-Saturday (ICC): .15 (-.09, .41) Saturday-Sunday (ICC): .37 (.07,.61)  Walking time per day: Friday: 60±40 mins Saturday: 20±24 mins Sunday: 17± mins  Numbers of walking episodes per day: Friday: 137±107 Saturday: 30±34 Sunday: 28±31  Number of steps per day:  Friday 2993±2314 Saturday 913±1402 Sunday: 703±922  Significant differences found between Friday, Saturday and Sunday for time spent walking, number of walking episodes and step count per day (p<.001). |  |
| Bongartz [30] | Mild-moderate dementia | **Volume:** Walk time (mins): 106.1±111.3 mins via PAMsys. 110.8±132.4 via uSense.   Walking episodes (n):  282.1±294.5 via PAMsys. 543.2±674.3 via uSense.  Steps (n): 5,325±5,767 via PAMsys 9,372±10,463 via uSense.  **Intensity** Average MET Score:  1.1±0.1 via PAMsys 1.6±0.2 via uSense. |  |
| Buckley [64] | Mild Alzheimer's disease | **Pattern:** Statistical Parametric Mapping time series analysis of walk time per day, bouts and steps per day, mean bout length, variability. Outcomes detected hourly over 24 hours and averaged across seven days. No differences were found between AD and controls. |  |
| Cerff [40] | Parkinson's MCI and dementia | **Volume:  % of Time Spent in activity** PD-NC: 0.21 (.03-.41)% PD-MCI: 0.21 (.08-.39)% PDD: .09 (.01-.26)%  % Time walking: PD-NC: 5.2% PD-MCI: 3.9% PDD: 1.8%  Activity bouts (n) PD-NC: 1,555 (58-6,385) PD-MCI: 1,470 (364-5,011) PDD: 859 (49-2,329)  Steps (n/day): PD-NC: 5,778 (43-14,164) PD-MCI: 4,742 (167-10,181) PDD: 2,362 (15-13,346**)  Intensity** Total Movement Intensity (g): PD-NC: .03 (.02-.06) PD-MCI: .03 (.01-.06) PDD: .03 (.006-.04)  Light Intensity (%): PD-NC: .08 (.02-.33)% PD-MCI: .06 (.02-.24)% PDD: .05 (0-.41)%  Moderate activity (%): PD-NC: .07 (.02-.15)% PD-MCI: .06 (.01-.13)% PDD: .02 (0-.07)%  Vigorous activity (%): PD-NC: .0008 (0-.03)% PD-MCI: .0007 (0-.03)% PDD: .0001 (0-.04)%  Activity related energy expenditure (MJ/Day): PD-NC: 832 (514-1,477)MJ/Day PD-MCI: 775 (202-1,081)MJ/Day PDD: 664 (314-1,205)MJ/Day  Total Energy Expenditure(MJ/day): PD-NC: 2,621 (1,898-3,500)MJ/Day PD-MCI: 2,572 (1,127-3,182)MJ/Day PDD: 2,306 (1,899-3,186)MJ/Day  Physical Activity ratio (relative EE to BMR of activity): PD-NC: 2.29 (1.35-3.13) PD-MCI: 2.16 (1.43-2.87) PDD: 2.20 (1.39-3.08)  Physical activity level (relative EE to BMR for all behaviours): PD-NC: 1.47 (1.32-1.90) PD-MCI: 1.48 (1.22-1.80) PDD: 1.44 (1.19-1.67)  **Pattern:** Mean bout length (s): PD-NC: 8.50 (0-41.24)s PD-MCI: 11.03 (6.06-23.91)s PDD: 12.02 (7.22-19.84)s | Cognitive status: No significant differences between any group for all HPA measures |
| Chang [46] | MCI | **Volume** Steps per day: 6,935.9±3,902 Distance per day (km): 4.6±2.8km  **Intensity:** Physical energy expenditure per day (kcal): 114.0±73.0 | Sex: Men have higher energy expenditure than women (p=.035)  Cognitive Status: No difference in steps, distance, or energy expenditure in early- or late-stage MCI. |
| David [31] | Alzheimer's disease | **Pattern** Daytime Mean Motor activity*: 168.1±30.05 activity counts  Night-time Mean Motor Activity: 25.82±11.79  *mean of all 1-minute activity count epochs | Neuropsychiatric: Daytime mean motor activity lower in group with apathy |
| Del Din [44] | MCI | **Volume:** Intervention Group 1: Walk time per day ( min): OF: 128.6±58.3 MCI: 125.2±48.7 PD: 117.3±59.1  % walking time: OF: 8.9±4.1 MCI: 8.7±3.4 PD: 8.2±4.1  Steps per day: OF: 9,833±4, 793 MCI: 9,354±3,712 PD: 8,874±4,538  Bouts per day:  OF: 264±105± MCI: 268±98 PD: 233±106  Intervention Group 2: Walk time per day ( min) OF: 142.2±59.9 MCI: 113.4±41.2 PD: 17.8±59.1  % walking time: OF: 9.9±4.2 MCI: 7.9±2.9 PD: 8.2±4.3  Steps per day: OF: 10,802±4,700 MCI: 8,252±3,218 PD: 9,000±4,535  Bouts per day:  OF: 288±97 MCI: 261±84 PD: 242±110  **Variability** Intervention Group 1: Variability (S2): OF: .62±.095 MCI: .605±.082 PD: .630±.094  Intervention Group 2: Variability (S2): OF: .612±.091 MCI: .562±.063 PD: .606±83  **Pattern** Intervention Group 1: Mean bout length:  OF: 28.9±5.7 MCI: 27.8±4.4 PD: 29.8±5.8  Alpha OF: 2.621±.27 MCI: 2.650±.23 PD: 2.553±.299  Intervention Group 2: Mean bout length:  OF: 29.2±6.0 MCI: 25.9±3.1 PD: 28.4±4.9  Alpha OF: 2.555±.209 MCI: 2.690±.237 PD: 2.587±.274 | Subgroup: All volume characteristics were lower in people with MCI and PD compared to older fallers (p≤.012) |
| Doi [53] | MCI | **Intensity** Light HPA (min/day): NS-WML: 353.6 ± 96.0 S-WML: 324.4±96.7  MVPA (mins/day): NS-WML: 24.1±18.7 S-WML: 18.6±17.5 | White matter lesions: People with severe WML spent less time in light HPA and MVPA compared to non-severe WML. |
| Eckert [63] | Mild-moderate dementia | **Volume** Steps per day (median): 2,662.5 (IQR: 1,175.1-4,686.5) |  |
| Falck [45] | MCI | **Volume:** Mean % of day spent in PA:  Controls: 12.07±7.19 MCI: 8.69±5.45  **Intensity:** Mean 10+ minute bouts per day:  Controls: 1.20±1.45 MCI: .40±.36 | Cognitive status: Controlling for age and sex, MCI had lower % of daily HPA (p=.030) and took fewer 10+ minute bouts/day compared to controls (p=.005). |
| Finnanger [55] | Dementia | **Intensity:** % of time in light activity: FB: 50.53±8.87% (32.9-65.66) DC: 50.19±11.48% (24.38-76.86)  % time in moderate activity: FB: 9.75±9.34% (0.17-38.43) DC: 6.29±5.97 (0.03-28.35) | Type of care: no differences between types of care for activity. |
| Harada [39] | Global cognitive impairment | **Volume:** Steps per day: 6,654.6±2,958.8 (range: 1855-21424.9)  Outdoor time (hours/day): 3:37:10±2:07:32 (range: 0:31:24-11:27:52) |  |
| Harada [48] | Global cognitive impairment | **Volume:** Steps per day: 6,721±2,975 (range: 1,676-21,424)  **Intensity:** MVPA per day: 31.7±21.1 mins (range: 1.7-154.7) |  |
| Hartman [50] | Dementia | **Volume:** CPM per day: Controls: 226± CPM Dementia: 186±76 CPM  **Intensity:** Very light HPA (Hours/% of measured time): Controls: 2.2 hours (1.9-2.6)/ 15% (12-16) Dementia: 2.3 hours (1.7-2.9)/16% (12-19)  Light-moderate HPA (Hours/% of measured time): Controls: 3.5 hours (2.7-4)/ 22% (18-25) Dementia: 2.7 hours (2-3.7)/20% (15-23)  MVPA (Hours/% of measured time): Controls: 1.5 hours (0.8-2)/ 10% (5-13) Dementia: 0.8 hours (0.4-1.5)/5% (2-10) | Cognitive status: Dementia group have lower CPM/day, light-moderate activity and MVPA compared to controls.   Age: No differences between dementia 80+ and dementia<80 for PA, differences found in these age groups for controls - younger group had higher CPM/day, light-moderate HPA and MVPA. |
| Hausdorff [61] | MCI | **Intensity:** Hours per day in ≥60 second walking bouts:  Controls: 1.05±0.66 hours MCI: 0.74±0.48 hours | Cognitive Status: MCI group spend less time waling in ≥60 second bouts than controls (p=.016)  Subtype: Based on MoCA sub scores, no differences between subgroups. |
| Hayes [32] | MCI | **Volume:** Mean CPM:  Controls: 0.70±0.15 MCI: 0.79±0.14  **Variability:** CoV daily activity: Controls: 0.45±.027 MCI: 0.44±.012  **Pattern:** Mean 24-hour wavelet variance (average of 6 consecutive 4-week measures):  Controls: 3.79±0.23 MCI: 4.07±0.14 | Cognitive status: MCI trended to be more active than controls. 24-hour wavelet variance was higher in MCI compared to controls (p=.008). |
| Hooghiemstra [59] | Mild-moderate early onset dementia | **Variability:** Intra-daily variability (higher values = more fragmented rhythm) EOD: .46±.16 Controls: .39±.10  Inter-daily stability (higher values= more stable rhythm) EOD: .79±.10 Controls: .81±.10 **Pattern:** Relative amplitude (higher values reflect greater amplitude and longer rhythm) EOD: .68±.15 Controls: .72±.12 | Cognitive status: EOD had higher IV compared to controls (p=.03) i.e., greater fragmentation of rest activity rhythm. |
| Iwata [42] | Dementia | **Volume:** Exercise/day (walking): Controls: 3.195±.4404 Dementia: 1.142±.2801  Female Controls: 1.912±0.31 Female dementia: .293±.08  Male Controls: 2.792±.045 Male Dementia: 1.772±.069  **Intensity:** Kcal/min during walking. Female Controls: .187±.03 Female Dementia .054±.01  Male Controls: .36±.04 Male Dementia .282±.04 | Cognitive status: People with dementia walked significantly less than controls (p=.0006). Female dementia participants walked less than female controls (p<.0001) but not the case in males. Both male and female dementia participants burned less calories per min than their male and female control counterparts (p <.006)  Gender: Females showed trends of less activity than males - but not significant. |
| James [35] | Dementia | **Volume:** Daily Physical activity: Dementia: 2.11x10^5^ activity count per day non-dementia: 3.07x10^5^ activity counts | Cognitive status: People with dementia had lower daily physical activity than non-dementia (p<.001). |
| Kwan [41] | MCI | **Volume:** Walk time per day (median): 149.8 (IQR: 77.6) mins Median step count per day: 12,256 (IQR: 4,540)  **Intensity** Median brisk walking time per day: 2.6 (IQR: 4.8) mins 1 minute peak cadence: 118 steps/min (IQR: 20.5) Median MVPA mins/week: 23 (IQR: 85) Median MVPA mins/day: 9 (IQR: 22) |  |
| Lu [57] | a-MCI and Alzheimer's disease | **Pattern:** Vector magnitude CPM during waking hours (6am-6pm; model corrected for age, gender, and wear time). Controls: 1,953.5±34.5 Low MoCA: 1,975.6±36.7 MCI: 2,073.2±56.7 AD: 1592.1±43.9  VM CPM during sleeping hours (6pm-6am; Model corrected for age, gender, and wear time): Controls: 223.6±7.6 Low MOCA: 217.6±7.9 MCI: 241.2±12.3 AD: 244.8±9.4 | Subgroups: VM CPM in each group peaked in the morning and declined gradually with a slight increase in the afternoon.   AD initiated their activity progressively later in the morning, and their activity peak was not as steep as the other groups, nor never met their threshold.  At night, activity patterns were similar across all groups.   Cognitive status: when adjusting for age, gender and wear time, AD participants had significantly lower VM CPM during the day (p<.05) compared to all other groups. |
| Mahlberg and Walther [51] | Alzheimer's disease | **Volume:** 24-hr actigraphic activity (counts per hour) Intervention AD: 76.1 (42.3-250.5) Placebo AD: 111.4 (29.3-258.5) Young Controls: 179.2 (97.1-288) Older Controls: 120.4 (26.3-328.7)  **Pattern:** Nocturnal actigraphic activity (Counts per hour) Intervention AD: 35.2 (19.9-80.1) Placebo AD: 20.5 (4.1-160.6) Younger Controls: 52.2 (12.5-111.7) Older Controls: 31.4 (4.3-87.7) |  |
| Mc Ardle [15] | Mild Alzheimer's disease | **Volume:** Walk time per day (mins): 214±66 Steps per day: 13,268±2,791 Daily bouts: 707±233  **Pattern** Mean bout length (secs): 17±4 Alpha: 1.627±.065  **Variability** Variability of bout length: .829±.059 |  |
| Mc Ardle [38] | Mixed cognitive impairment (MCI and dementia) due to Alzheimer's disease, dementia with Lewy bodies and Parkinson's disease dementia | **Volume:** Walk time (mins/day) Controls: 196±63 Cognitive impairment: 148±64 AD: 168±55 DLB: 147±72 PDD: 106±45  Steps per day Controls: 14,204±4,817 CI: 10,172±4,617 AD: 11,425±4,172 DLB: 10,197±5229 PDD: 7,305±3018)  Bouts per day Controls: 630±166 CI: 566±209 AD: 615±211 DLB 565±213 PDD 459±159  % of day spent walking Controls: 13±4 CI: 10±4 AD: 12±4 DLB 10±5 PDD 7±3  **Pattern:** Mean bout length (secs) Controls: 19±4 CI: 16±3 AD: 17±4 DLB: 15±3 PDD: 14±3  Alpha (ratio long: short bouts) Controls: 1.611±.038 CI: 1.640±.067 AD: 1.622±.045 DLB: 1.640±.054 PDD: 1.678±.108  **Variability:** Variability of bout length Controls: .882±.083 CI: .819±.081 AD: .835±.081 DLB: .820±.068 PDD: .780±.093 | Cognitive status: controlling for age and sex, CI walked less, took less steps and spent less % of their day walking compared to controls. They also had shorter bout lengths and lower variability compared to controls (P<.001)  Subtype: controlling for age and gender, both DLB and PDD walked less, took less steps and spent a lower % of their day in walking than controls p<.003). They also had shorter bout lengths and less variability than controls. PDD also showed higher alpha scores (p<.01).  PDD also demonstrated shorter bout lengths, lower variability, and higher alpha than AD. They also had higher alpha than DLB |
| Nickerson and Shade [47] | MCI | **Volume:** Steps: 5,317.35±2,699.9 (19-16,271) |  |
| Pedroso [49] | Alzheimer's disease | **Volume:** Steps/day Controls: 6,154 (4,317-8,556) AD: 1,509 (605-4,411) | Cognitive status: AD took less steps compared to controls (p<.01). |
| Rackoll [34] | MCI | **Volume**  Average activity count per day Controls: 426±139 MCI: 396±169  **Intensity** Active energy expenditure kcal/day Controls: 412±176 MCI: 333±265  % time in light activity Controls: 23%±6 MCI: 25±5%  % time in Moderate activity:  Controls: 2±1% MCI: 3±4%  % time in Vigorous activity:  Controls 0±0 MCI: 0±0  % time in MVPA:  Controls: 3±2% MCI 3±4%  **Pattern:** MCI were less active than controls in morning hours between 5.40 and 6.20 (p=.031) and in the evenings between 0.05-10.25 (p=.001). | Cognitive status: activity levels similar between groups, no significant difference for intensity of activity.  Variability higher in MCI participants compared to controls - higher activity levels throughout waking hours with a slight decrease below levels of controls in the evening hours. |
| Rawtaer [43] | MCI | **Volume:** Steps per day:  Controls: 4,033±2,148 MCI: 3,407±2,688 | Cognitive status: No significant difference between AD and controls for steps (p=.40) |
| Schwenk [37] | Dementia | **Volume:** % of time walking/24 hours: Fallers: 4.1±3.1% Non-fallers: 4.9±2.8%  **Variability:** Walking bout duration variability (CV%): Fallers: 87.1±35.5% Non-fallers: 126.5±80.1%  **Pattern:** Mean bout length: Fallers: 10.7±2.3 secs Non-fallers: 13.5±5.2 secs  Longest bout length duration: Fallers: 89.9±100.2 secs Non-fallers: 200.5±281.7 secs | Faller Status: Non-fallers took longer walking bouts (p=.008), had a longer longest walking bout duration (p=.009) and had greater variability between walking bout durations (p=.027) |
| Taylor [56] | Mild-moderate dementia | **Volume:** Steps per day:  Dementia: 3,307 (1,633-4,923) Controls: 5,526 (4,037-7,192)  Walking bouts per day: Dementia: 250 (155-360) Controls: 346 (284-457)  Mean daily walk time (mins):  Dementia: 43 (25-64) Controls: 63 (46-84)  **Intensity** Number of walks >60 seconds: Dementia: 10.3 (4.8-19.8) Controls: 14.4 (10.1-22-5) | Cognitive status: People with dementia take less steps and walking bout per day, have shorter daily walk time (p<.001 for all), and take less walking bouts 60+ seconds compared to controls (p=.044)  Faller status: Fallers take lower walking bouts per day (median: 210) compared to non-fallers (median: 306), spend less time walking per day (33.6 mins vs 58.2 mins). No difference in steps per day or other characteristics. |
| van Alphen [33] | Dementia:  Alzheimer's disease: 48.6% Vascular dementia: 16.2% Alzheimer's with vascular problems: 10.8% Dementia with Lewy bodies: 8.1% Frontotemporal dementia: 2.7% Parkinson's disease dementia: 5.4% Korsakoff dementia: 2.7% Unspecified dementia: 5.4% | **Volume:** Total daily HPA (Counts per day): Community dwellers with dementia, all: 2.21±1.26 (x10^5) AD: 2.38±1.11 (x10^5) Other dementia subtypes: 1.97±1.44 (x10^5)  Controls: 2.82±0.83 (x10^5)  Nursing home dementia, all: 1.69±1.33 (x10^5) AD: 1.86±1.54 (x10 ^5) Other dementia; 1.45±0.93 (x10^5)  **Pattern** Community-dwellers were more active than nursing home residents between 8am-6pm, and from 10-11pm. Community dwellers were most active between 9-10am, and 2-3pm.  Community dwellers with dementia were significantly less active than controls between 11am-1pm. | Residence status: Total daily HPA In institutionalised participants was 23.5% lower than community dwellers (p=.004). Community dwellers spent more time is 0-99 CPM and 400-499 CPM and 700-799 CPM intensities of activity.   Cognitive status: Community dwellers with dementia had 21.6% lower daily HPA levels than controls (p=.007). Controls spent more time in higher activity ranges, specifically 500-900 CPM compared to community dwellers with dementia.   Subtype: No difference between AD and non-AD, although AD participants were 25.5% more activity - no interaction for living situation or dementia type.   Gender: no significant effect for gender. |
| Varma and Watts [52] | Alzheimer's disease | **Volume:** Total HPA (vm): AD: 378,110±32,004.3 Controls: 514,326.3±25,877.24  **Intensity:** Low-intensity HPA (AD-Controls; vm): -39,538.380   Moderate-intensity HPA (AD-Controls; log vm): -.679  **Variability:** RMSD (AD-controls): 119.873  **Pattern:**  Peak activity (AD-Controls; vm): -753.129  Time of peak activity (AD-controls; mins): 59.877  Morning activity (7am-12pm; difference AD-Controls) Moderate HPA (log vm): -.566 Peak Activity: -625.736 RMSD: -152.215  Afternoon (12pm-5pm; difference AD-controls) Moderate PA: -.43 Peak activity: -389.725 RMSD: -76.556  Evening (5-10pm; difference AD-Controls) Moderate PA: -.329 Peaking activity: -104.200 RMSD: -63.63 | Cognitive status: People with AD are less active than controls (p<.01), spend less time in moderate intensity PA, have lower peak activity (vm; p=.006) and Root mean square difference (.020). AD had lower moderate HPA (p=.028), peak activity (p=.023) and RMSD (p=.037) in the mornings, lower peak activity (p=.047) in the afternoon, and no differences in the evening than controls. |
| Watts [62] | Mild Alzheimer's disease | **Volume:** Average vector magnitude (AD vs controls, controlling for day of wear, age, education, sex, cardiorespiratory capacity): AD is 20.55% less physically active on average.   **Variability:** Intra-individual variability (AD vs controls, controlling for day of wear, age, education, sex, cardiorespiratory capacity): AD is 0.54% more variable than controls. |  |

Abbreviations: ICC = intra-correlation co-efficient, MET = metabolic equivalent, PD = Parkinson’s disease, NC =normal cognition, MCI = mild cognitive impairment, PDD = Parkinson’s disease dementia, HPA = habitual physical activity, EE = energy expenditure, BMR = basal metabolic rate, OF = older fallers, NS-WML: Not significant white matter lesions, S-WML = significant white matter lesions, MVPA = moderate-vigorous physical activity, PA = physical activity, FB: Farm-based dementia care, DC = day-care, CPM = counts per minute, MoCA = Montreal Cognitive Assessment, CoV = coefficient of variance, EOD = early onset dementia, VM = vector magnitude, AD = Alzheimer’s disease, CI = cognitive impairment, DLB = dementia with Lewy bodies, RMSD = Root mean square difference,
